# Genomic Profile of Chronic Lymphocytic Leukemia in Korea Identified by Targeted Sequencing

**DOI:** 10.1371/journal.pone.0167641

**Published:** 2016-12-13

**Authors:** Jung-Ah Kim, Byungjin Hwang, Si Nae Park, Sunghoon Huh, Kyongok Im, Sungbin Choi, Hye Yoon Chung, JooRyung Huh, Eul-Ju Seo, Je-Hwan Lee, Duhee Bang, Dong Soon Lee

**Affiliations:** 1 Department of Laboratory Medicine, Seoul National University College of Medicine, Seoul, Korea; 2 Department of Chemistry, Yonsei University, Seoul, Korea; 3 Cancer Research Institute, Seoul National University College of Medicine, Seoul, Korea; 4 Bachelor of Science, University of British Columbia, Vancouver, Canada; 5 Department of Pathology, Asan Medical Center, University of Ulsan College of Medicine, Seoul, Korea; 6 Department of Laboratory Medicine, Asan Medical Center, University of Ulsan College of Medicine, Seoul, Korea; 7 Department of Internal Medicine, Asan Medical Center, University of Ulsan College of Medicine, Seoul, Korea; University of Manitoba, CANADA

## Abstract

Chronic lymphocytic leukemia (CLL) is extremely rare in Asian countries and there has been one report on genetic changes for 5 genes (*TP53*, *SF3B1*, *NOTCH1*, *MYD88*, and *BIRC3*) by Sanger sequencing in Chinese CLL. Yet studies of CLL in Asian countries using Next generation sequencing have not been reported. We aimed to characterize the genomic profiles of Korean CLL and to find out ethnic differences in somatic mutations with prognostic implications. We performed targeted sequencing for 87 gene panel using next-generation sequencing along with G-banding and fluorescent in situ hybridization (FISH) for chromosome 12, 13q14.3 deletion, 17p13 deletion, and 11q22 deletion. Overall, 36 out of 48 patients (75%) harbored at least one mutation and mean number of mutation per patient was 1.6 (range 0–6). Aberrant karyotypes were observed in 30.4% by G-banding and 66.7% by FISH. Most recurrent mutation (>10% frequency) was *ATM* (20.8%) followed by *TP53* (14.6%), *SF3B1* (10.4%), *KLHL6* (8.3%), and *BCOR* (6.25%). Mutations of *MYD88* was associated with moderate adverse prognosis by multiple comparisons (*P* = 0.055). Mutation frequencies of *MYD88*, *SAMHD1*, *EGR2*, *DDX3X*, *ZMYM3*, and *MED12* showed similar incidence with Caucasians, while mutation frequencies of *ATM*, *TP53*, *KLHL6*, *BCOR* and *CDKN2A* tend to be higher in Koreans than in Caucasians. Especially, *ATM* mutation showed 1.5 fold higher incidence than Caucasians, while mutation frequencies of *SF3B1*, *NOTCH1*, *CHD2* and *POT1* tend to be lower in Koreans than in Caucasians. However, mutation frequencies between Caucasians and Koreans were not significantly different statistically, probably due to low number of patients. Collectively, mutational profile and adverse prognostic genes in Korean CLL were different from those of Caucasians, suggesting an ethnic difference, while profile of cytogenetic aberrations was similar to those of Caucasians.

## Introduction

Chronic lymphocytic leukemia/small lymphocytic leukemia (CLL/SLL) is a clonal B cell proliferative disorder. CLL is a typical malignancy that displays ethnic differences: Although it is one of the most common leukemia in Caucasians, it is extremely rare in Asian countries [1, 2]. While the incidence rate (per 100,000 person-years) of CLL is 3.83 in Caucasians [2], it is only 0.04 in Korea [3], 0.48 in Japan [4], 0.6 in Koreans residing in America, and 0.8 in Chinese residing in America [5]. These data indicate that the incidence rate of CLL is 10- to 20-fold higher in Caucasians than in Eastern populations. Ethnic differences are observed in the median age of disease development as well. The median age at disease diagnosis is 70 year in the Caucasians, while the median age of in Asian CLL is 61 year [6]. This type of ethnic difference is also observed in myelodysplastic syndrome (MDS), which has a mean age of development of 71 years in the West and 57 years in Asia [7]. The causes of these ethnic differences might be related to the etiology of CLL.

CLL exhibits marked genetic heterogeneity, with a relatively large number of genes showing recurrent mutations. Mutations in the *SF3B1*, *ATM*, *TP53*, and *NOTCH1* genes were the most frequently involved recurrent mutations [8, 9]. On the other hand, a study on Chinese CLL patients that examined the frequencies of *TP53*, *SF3B1*, *NOTCH1*, *MYD88*, and *BIRC3* using Sanger sequencing reported an ethnic difference between Caucasian and Chinese patients [10]. The frequency of *TP53* (15%), and *SF3B1* (5%) in Chinese CLL were strikingly different from those reported in Caucasians. *TP53* mutation is more common in Chinese CLL than in the Caucasian CLL, while *SF3B1* mutation is less common in Chinese CLL.

Among these various gene mutations associated with CLL, mutations in *TP53*, *ATM*, *NOTCH1*, and *SF3B1* have been reported to be significantly related to poor prognosis in Caucasians [11]. The *TP53* mutation is notoriously related to adverse survival outcomes and drug resistance [12, 13], and the *ATM* mutation is associated with rapid disease progression [14], while the *NOTCH1* mutation is an independent poor prognostic factor [15]. The *SF3B1* mutation is also associated with rapid progression and adverse survival outcomes in CLL [16]. Among these mutations, only *TP53* and *NOTCH1* mutations were found to be poor prognosis factors in Chinese patients, while *ATM* and *SF3B1* mutations were not [10].

With regards to ethnic difference in disease incidence, differences in single-nucleotide variants (SNVs) have been reported between Caucasian and Asian populations [17, 18]. Of note, 6p25.3 in the *IRF4* gene region, 2q37.1 in the *SP140* gene region and 2q13 in the *ACOXL* gene region has been associated with ethnic differences. Change in life style including westernized diet and an absolute increase of elderly population will affect the incidence of CLL in Asian countries. We also reported the increasing incidence of CLL in Korea, based on the database of Korean National Cancer Registry [19]. However, genome-wide profile of patients with CLL have not yet been reported in Korea and in Asian. To investigate whether genetic mutations and the prognostic impact of known adverse mutations differ between Koreans and Caucasian patients with CLL, we performed target-capture sequencing of 87 hematologic malignancy-related genes. To the best of our knowledge, this study provides the first comprehensive mutation analysis of Asian patients with CLL using NGS.

## Materials and Methods

### Study populations

A total of 71 patients diagnosed with CLL/SLL between September 2001 and October 2013 at Seoul National University Hospital (SNUH, n = 58) and Asan Medical Center (n = 13) were enrolled. All of the patients were Korean. The diagnosis of CLL/SLL was based on the World Health Organization (WHO, 2008) classification criteria [20] and the 2008 International Workshop on Chronic Lymphocytic Leukemia-National Cancer Institute criteria (IWCLL-NCI) [21]. Fluorescence in situ hybridization (FISH) for IgH/CCND1 translocations was performed to confirm that the disease was not a leukemic phase of mantle cell lymphoma. Clinical staging was performed using the Binet staging system (classes A, B and C) [21]. Laboratory data including age, sex, diagnosis and therapy start date, complete blood count, and bone marrow (BM) pathology were reviewed. All BM and lymph node samples were collected with informed consent, and the study was approved by the Institutional Review Board of SNUH (1307-090-505). Participants provide their written informed consent to participate in this study.

### Bone marrow examination and Leukemia-lymphoma marker study

Hematopathologists reviewed the Wright-stained BM smears and hematoxylin and eosin—stained sections of BM trephine biopsies to determine the percentages and patterns of BM infiltration by lymphocytes. The median lymphoid cell percentage was 70% (range, 5–95%). The median BM cellularity was 65% (range, 15–95%). The leukemia-lymphoma marker study (TdT, CD2, CD3, CD5, CD7, CD10, CD19, CD20, CD22, CD23, FMC7, CD45, CytoCD3, CD56, Kappa, and Lambda (BD Biosciences, San Jose, CA, USA) was performed. Six patients were negative for CD5, being categorized as atypical CLL. ZAP-70 immunohistochemical staining (Cell Marque, Rocklin, CA, USA) was performed on BM section.

### G-banding and Fluorescence *in situ* hybridization

For G-banding, B cell-mitogen, tetradecanoylphorbol acetate (TPA; phorbol-12-myristate-13-acetate) was added with subsequent culture for 4 days. FISH for enumeration of chromosome 12 and for detection of 13q14.3 deletion, 17p13 deletion, 11q22 deletion and *IgH/CCND1* translocations (to exclude mantle cell lymphoma) was performed: the LSI D13S319/LSI13q34/CEP12 Multi-color Probe, LSI *TP53* (17p13.1) SpectrumOrange Probe, Vysis *IGH/CCND1* XT DF FISH Probe (all from Abbott Molecular/Vysis, Des Plaines, IL, USA), and XL *ATM/TP53* Probe (Metasystems, GmbH, Altlussheim, Germany). Interphase FISH was performed on stored BM nuclear cells according to the manufacturer’s instruction. The cut-off values for the deletion, amplification, or translocation of chromosomal regions were based on the mean ±3 standard deviations of the normal controls (n = 20). Among 71 patients, we applied 2 different set of reference ranges since we had reset the reference ranges during study period (from patient 1–11: 1.06% for trisomy 12, 4.58% for 13q14.3 deletion, 7.39% for 17p13 deletion, 5.59% for 11q22 deletion/from patients 12–71: 1.5% for trisomy 12, 4.01% for 13q14.3 deletion, 1.7% for 17p13 deletion, 5% for 11q22 deletion).

### Targeted sequencing

To gain insight into the genetic lesions that drive CLL, we performed targeted sequencing for 87 hematology malignancy-related genes (S1 Table). Using Agilent 2200 TapeStation system (Santa Clara, CA, USA), we performed quality control (QC) of the input material for subsequent library preparation and hybridization capture step. If the DIN (DNA Integrity Number, provided in the instrument’s internal algorithm) value was low, we did not further process DNA because it was highly degraded and these low-quality DNAs cannot be used for library preparation step. We collected 71 patients’ samples, but 19 samples’ DIN value were low, so only 48 samples were sequenced. And we did not purified tumor cells only, but the mean percentage of CD20+ B lymphocytes was 70% (n = 48), we assumed tumor cells are included as those percentages. Besides, we used THetA (Tumor Heterogeneity Analysis) [22] to calculate the proportion of cancer cells in the admixture. The tumor purity of CLL samples ranged from 80 to 95%.

**Fig 1:**
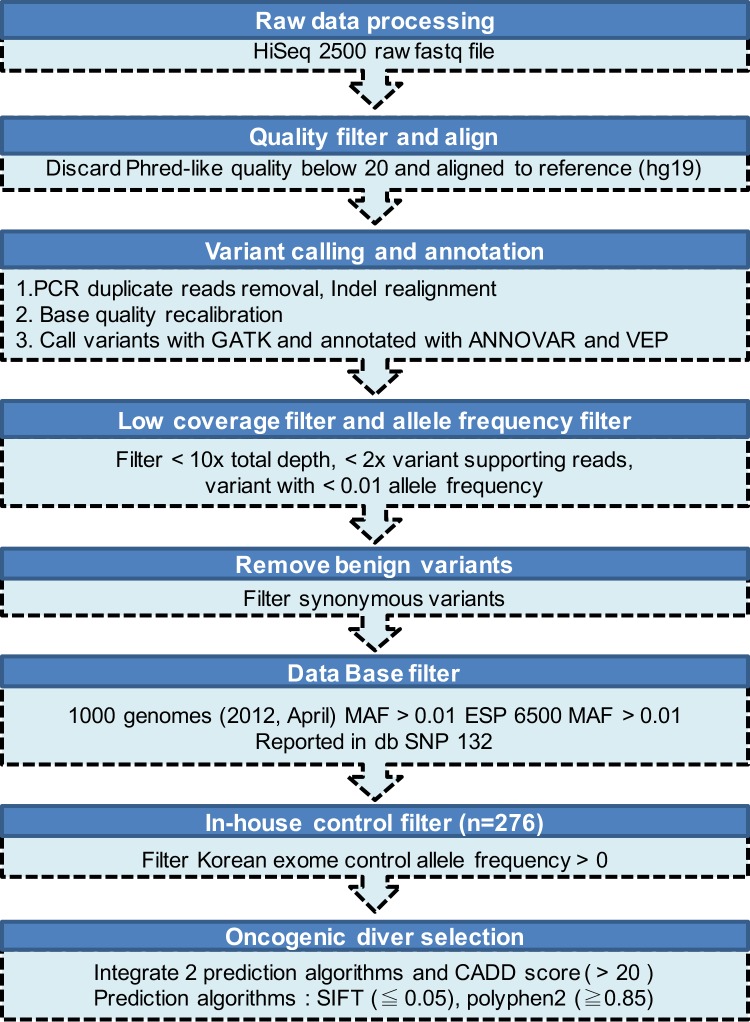
Summary of the variant-filtering pipeline.

gDNA shearing to generate the standard library and the hybridization step targeting only exonic regions were performed by Celemics Inc. (Seoul, Korea). Briefly, ~ 500 ng of sequencing library was denatured at 95°C for 5 min and then incubated at 65°C before addition of the customized-baitset reagent and Cot, Salmon sperm and adaptor-specific blocker DNA in hybridization buffer. After 24-h incubation, the library was captured on T1 Magnetic Beads and off-target library was washed. Then, the target captured library was amplified (16 cycles). After amplification, the samples were purified using AMPure XP Beads. The final quality was assessed using the Agilent 2200 TapeStation System (Santa Clara, CA, USA). We sequenced a total target length of 259-kb regions using the paired-end 150-bp rapid-run sequencing mode on an Illumina HiSeq 2500 platform. The mean sequencing depth for the targeted regions (259 kb) was 231-fold (n = 48). Because a matched control sample was not included in this study, we applied a stringent variant selection pipeline to prioritize the high-confidence set of somatic mutations (Fig 1).

### Analysis of Illumina sequencing and variant prioritization

First, we trimmed raw fastq files with bases with Phred-like quality below 20. Then, data was mapped to the reference genome (hg19) with Burrows-Wheeler Aligner (BWA, v0.6.2). PCR duplicate read was removed using Picard 1.98. Indel realignment and base quality recalibration was performed using GATK (v.2.7–2). Then, single nucleotide variants and indels were called using “HaplotypeCaller” module in GATK was used which is based on local re-assembly of potential variant regions combined with likelihood estimation of candidate haplotypes. We annotated variants with ANNOVAR and VEP. Further, we retain variants if they were found in >2 reads among >10 total reads. Benign mutations (Synonymous mutation) were further removed. Moreover, two additional control sets were applied to discard possible polymorphisms (variants with a frequency less than 1% in the 1000genomes as well as ESP6500 database). Known SNPs in private database dbSNP132 were also removed. Finally, we discarded variants presented in in-house healthy Korean exome controls (n = 276) (strict cutoff based on very rare incidence of CLL in Korea (0.13 /100,000)) [23].

We retained variants in the COSMIC (v68) database and known somatic mutations by exhaustively searching PubMed database to confirm somatic origin. Additionally, we manually inspected error-prone mapping regions and removed potential false positive variant and regions with highly repetitive sequences were removed.

### Oncogenic driver selection

After filtering low confidence polymorphisms, we further narrowed down remaining potential pathogenic candidate mutations using known functional amino acid change prediction algorithms (SIFT [24], PolyPhen-2 [25]). Also, we scored all variants with CADD [26] (URL: http://cadd.gs.washington.edu/home) algorithm and prioritized variants based on the scaled Phred-like c-score. This algorithm uses machine-learning models to distinguish deleterious variants from neutral ones. Lastly, we chose final variant set based on the scheme as follows:

1. Retain if more than 2 algorithm predicted as damaging (prediction results for SIFT and Polyphen-2 as “Damaging or Probably damaging” and classified CADD c-score > 20 as damaging)
2. Alternatively, when one of the 3 predictions is annotated as NA (not available), we rescued if damaging in one (1/2) or two algorithms (2/2). When 2 of the 3 prediction algorithms are annotated as NA, we rescued variants if remaining one algorithm predicted as damaging (1/1).

Hereafter, mutated genes refer to those with any variant that has passed the above criteria.

### Validation with Sanger sequencing

For validation of the targeted sequencing, we have selected random subset of mutation, *ATM*, *TP53*, *SF3B1*, *LAMB4* and *EZH2*. Primers (S2 Table) for PCR were designed from ±150 bp upstream and downstream of the target gene. The conditions for PCR were as follows: 1) 3 min of initial heating at 98°C followed by 20 cycles of 98°C 30 s, 60°C 30 s, 72°C 1 min and a final elongation step at 72°C for 10 min. Sequence analysis was performed using Lasergene 10/SeqMan 5.01 (DNASTAR Inc., USA).

Since the cost for validating all the mutations called by variant caller would be cost-demanding [27], we have selected sixteen random subset of mutations (in wide range of variant allele frequencies) for Sanger sequencing for validation purpose. All of these samples had mutations.

### Statistical analysis

Chi-squared test, Fisher’s exact test, Pearson's product-moment correlation, log-rank test, the Kaplan-Meier method, and the Cox proportional hazards model for differences between the survival curves and hazard ratios with 95% confidence interval (CI). Statistical analyses were performed using R software (version 3.3.0, http://www.r-project.org). False discovery rate (FDR) was applied in our study for multiple comparisons [28]. Raw *P*-values<0.05 were mentioned, and adjusted *P*-values<0.05 considered statistically significant.

## Results

### Patient characteristics

S3 Table summarizes the baseline clinical features of patients with CLL (n = 71). Their median age at diagnosis was 61 years (range, 23–81). The mean values of hemogram were 15,040 (x 10^9^/L) WBC (range 1,340–353,050), 11,248 (x 10^9^/L) lymphocytes (range 670–247,135), 12.8 (g/L) hemoglobin (range 7–17), and 163 (x 10^9^/L) platelets (range 41–389). A total of 64.8% (46/71) of the patients were in Binet stage A, 14.1% (10/71) in stage B and 21.1% (15/71) in stage C. Median follow-up period was 37 months, and the median overall survival was 112 months. 5-year overall survival rate was 63.2%, and 10-year overall survival rate was 49.4%. Eight percent of the patients (6/71) progressed to Richter’s syndrome; 4 patients exhibited transformation to diffuse large B cell lymphoma, 1 patient to prolymphocytic leukemia, and 1 patient to composite lymphoma of peripheral T cell lymphoma (unspecified) and large B cell lymphoma.

### Conventional karyotyping and fluorescence *in situ* hybridization

Cytogenetic abnormalities were examined through G-banding (60/71 patients) and FISH studies (51/71 patients). Of the 56 patients in whom karyotyping was successful, 30.4% (17/56) had aberrant karyotypes. FISH was able to detect cytogenetic aberrations in 34 patients (66.7%, 34/51) (S4 Table). Ten patients showed three or more chromosomal aberrations representing complex karyotypes. Deletion of 13q14 was detected in 45.8% (22/48 patients), followed by trisomy 12 in 30.0% (15/50 patients), deletion of 17p13 in 23.5% (12/51 patients), and deletion of 11q22 in 15.9% (7/44 patients). The frequency of 13q14 deletion and trisomy 12 were in the line with previous studies on Caucasian and Asian CLL patients, but the proportion of 17p13 deletion and 11q22 deletion were different. Twenty-two cases showed one abnormality (43.1%, 22/51), two abnormalities were observed in 13 cases (25.5%, 13/51), and three abnormalities were observed in 3 patients (5.9%, 5/51). In total, 8 patients (17.0%, 8/47) showed no cytogenetic aberration by both methods and 23 patients showed aberrations that were detected only by FISH (48.9%, 23/47).

### Gene mutations

A total of 6.6 million reads were obtained for each patient, and 98.9% of the reads mapped to the target region (S5 Table). Gene mutations were observed in 35 genes, and somatic mutations were detected in 71 different sites. Most of these mutation sites (84.5%, 60/71) are novel to the literature (Table 1). We observed that 75% (36/48) of the patients harbored at least one mutation, and an average of 1.6 mutations per patient was detected among the 48 patients (range 0–6). Among the 36 of 48 patients (75%) who carried at least one mutation, an average of 2.1 mutations were detected per patient (range 1–6). Among the 71 mutations, 49 were missense mutations, while 13 were frameshift mutations, and 4 were non-frameshift mutations; the remaining 5 were stop, gain or loss mutations. These somatic mutations were validated through Sanger sequencing. Sixteen randomly selected mutations were successfully confirmed (S2 Table).

**Table 1:** Somatic mutations found in 48 patients with CLL.

Chromosome	Gene	Position	Reference	Alternative	Mutation Type	AA Change	Patient No.
**1**	***ITPKB***	**226923392**	**G**	**A**	**nonsynonymous SNV**	**p.R590W**	**19**
**2**	***PRKD3***	**37543553**	**G**	**A**	**stopgain SNV**	**p.R39X**	**29**
**2**	***LRP1B***	**141459813**	**TCTC**	**-**	**frameshift deletion**	**p.2066_2067del**	**40**
**2**	***SF3B1***	**198266834**	**T**	**C**	**nonsynonymous SNV**	**p.K700E**	**48, 64, 65**
**2**	***SF3B1***	**198267361**	**T**	**C**	**nonsynonymous SNV**	**p.K666E**	**16**
**2**	***SF3B1***	**198267484**	**G**	**C**	**nonsynonymous SNV**	**p.R625G**	**25**
**3**	***MYD88***	**38182641**	**T**	**C**	**stoploss SNV**	**p.X160R**	**34, 62**
**3**	***GATA2***	**128205685**	**C**	**T**	**nonsynonymous SNV**	**p.A64T**	**16**
**3**	***KLHL6***	**183212036**	**T**	**C**	**nonsynonymous SNV**	**p.Y394C**	**8**
**3**	***KLHL6***	**183273170**	**G**	**A**	**nonsynonymous SNV**	**p.A91V**	**65**
**3**	***KLHL6***	**183273185**	**T**	**A**	**nonsynonymous SNV**	**p.H86L**	**6**
**3**	***KLHL6***	**183273188**	**C**	**T**	**nonsynonymous SNV**	**p.C85Y**	**23**
**3**	***KLHL6***	**183273189**	**A**	**G**	**nonsynonymous SNV**	**p.C85R**	**65**
**4**	***KIT***	**55604628**	**C**	**T**	**stopgain SNV**	**p.R946X**	**15**
**4**	***FAT4***	**126239804**	**ACAAGAATGG**	**-**	**frameshift deletion**	**p.746_749del**	**64**
**4**	***FAT4***	**126241813**	**A**	**T**	**nonsynonymous SNV**	**p.N1416I**	**64**
**4**	***FAT4***	**126373317**	**C**	**A**	**nonsynonymous SNV**	**p.R3716S**	**15**
**5**	***CSF1R***	**149457767**	**C**	**T**	**nonsynonymous SNV**	**p.V213M**	**35**
**6**	***BRD2***	**32945698**	**GAG**	**-**	**nonframeshift deletion**	**p.498_499del**	**8, 35**
**7**	***LAMB4***	**107703233**	**T**	**TA**	**frameshift insertion**	p.S1090*	**57**
**7**	***LAMB4***	**107706935**	**C**	**T**	**nonsynonymous SNV**	**p.G853S**	**8**
**7**	***LAMB4***	**107732794**	**G**	**A**	**nonsynonymous SNV**	**p.A513V**	**35**
**7**	***POT1***	**124499011**	**C**	**A**	**nonsynonymous SNV**	**p.K103N**	**28**
**7**	***EZH2***	**148506437**	**G**	**A**	**nonsynonymous SNV**	**p.A636V**	**41**
**7**	***EZH2***	**148514402**	**CAGCACCACTCCACTCCACATTCTCAG**	**-**	**nonframeshift deletion**	**p.388_397del**	**38**
**8**	***SCRIB***	**144889100**	**G**	**T**	**nonsynonymous SNV**	**p.D754E**	**42**
**9**	***CDKN2A***	**21974808**	**T**	**A**	**nonsynonymous SNV**	**p.S7C**	**60**
**9**	***NOTCH1***	**139390648**	**AG**	**-**	**frameshift deletion**	p.P2515*fs	**40, 63**
**9**	***NOTCH1***	**139390815**	**G**	**-**	**frameshift deletion**	**p.Q2459fs**	**43**
**9**	***NOTCH1***	**139402795**	**C**	**T**	**nonsynonymous SNV**	**p.G1072S**	**43**
**10**	***EGR2***	**64573332**	**C**	**T**	**nonsynonymous SNV**	**p.E356K**	**21**
**11**	***SF1***	**64534502**	**A**	**AGGC**	**nonframeshift insertion**	**p.484ins**	**65**
**11**	***ATM***	**108115594**	**C**	**T**	**stopgain SNV**	**p.R248X**	**57**
**11**	***ATM***	**108163400**	**A**	**T**	**nonsynonymous SNV**	**p.L1497F**	**64**
**11**	***ATM***	**108183152**	**A**	**T**	**nonsynonymous SNV**	**p.E1978V**	**5**
**11**	***ATM***	**108186757**	**G**	**A**	**nonsynonymous SNV**	**p.E2039K**	**48**
**11**	***ATM***	**108196912**	**T**	**C**	**nonsynonymous SNV**	**p.L2312P**	**21**
**11**	***ATM***	**108201089**	**C**	**G**	**nonsynonymous SNV**	**p.R2486G**	**63**
**11**	***ATM***	**108205790**	**AG**	**-**	**frameshift deletion**	**p.2702_2702del**	**21**
**11**	***ATM***	**108206579**	**A**	**G**	**nonsynonymous SNV**	**p.D2720G**	**42**
**11**	***ATM***	**108206581**	**G**	**A**	**nonsynonymous SNV**	**p.D2721N**	**64**
**11**	***ATM***	**108206666**	**A**	**T**	**nonsynonymous SNV**	**p.K2749I**	**19**
**11**	***ATM***	**108216576**	**C**	**T**	**nonsynonymous SNV**	**p.P2842L**	**31**
**11**	***ATM***	**108216601**	**G**	**T**	**nonsynonymous SNV**	**p.L2850F**	**26**
**11**	***ATM***	**108224508**	**A**	**C**	**nonsynonymous SNV**	**p.Q2896P**	**48**
**11**	***ATM***	**108235838**	**G**	**T**	**nonsynonymous SNV**	**p.W2960C**	**64**
**12**	***SH2B3***	**111856250**	**G**	**A**	**nonsynonymous SNV**	**p.E101K**	**31**
**12**	***SH2B3***	**111885306**	**T**	**TG**	**frameshift inserstion**	**p.398ins**	**38**
**13**	***RB1***	**49039195**	**C**	**T**	**nonsynonymous SNV**	**p.S758L**	**34**
**15**	***TCF12***	**57489989**	**A**	**G**	**nonsynonymous SNV**	**p.K182R**	**57**
**15**	***CHD2***	**93563337**	**C**	**T**	**nonsynonymous SNV**	**p.H1668Y**	**30**
**17**	***TP53***	**7576874**	**TCCAGTGGTTTCT**	**-**	**frameshift deletion**	**p.188_192del**	**20**
**17**	***TP53***	**7577097**	**C**	**T**	**nonsynonymous SNV**	**p.D149N**	**62**
**17**	***TP53***	**7577121**	**G**	**A**	**nonsynonymous SNV**	**p.R141C**	**16**
**17**	***TP53***	**7577511**	**A**	**G**	**nonsynonymous SNV**	**p.L125P**	**35**
**17**	***TP53***	**7577547**	**C**	**T**	**nonsynonymous SNV**	**p.G113D**	**33**
**17**	***TP53***	**7578190**	**T**	**C**	**nonsynonymous SNV**	**p.Y88C**	**33**
**17**	***TP53***	**7578208**	**T**	**C**	**nonsynonymous SNV**	**p.H82R**	**63**
**17**	***TP53***	**7578212**	**G**	**A**	**stopgain SNV**	**p.R81X**	**13**
**18**	***SETBP1***	**42643500**	**C**	**G**	**nonsynonymous SNV**	**p.P1543R**	**21**
**19**	***CEBPA***	**33792731**	**G**	**GGCGGGT**	**nonframeshift insertion**	**p.197_199ins**	**61**
**20**	***SAMHD1***	**35579956**	**T**	**-**	**frameshift deletion**	**p.D31fs**	**25**
**21**	***RUNX1***	**36259207**	**G**	**A**	**nonsynonymous SNV**	**p.P68L**	**51**
**X**	***ZRSR2***	**15818044**	**CT**	**-**	**frameshift deletion**	**p.57_58del**	**5**
**X**	***BCOR***	**39914637**	**A**	**-**	**frameshift deletion**	**p.M1523fs**	**43**
**X**	***BCOR***	**39923684**	**C**	**T**	**nonsynonymous SNV**	**p.R1118H**	**11**
**X**	***BCOR***	**39933386**	**CTGGGCACCTTCGC**	**-**	**frameshift deletion**	**p.400_405del**	**65**
**X**	***DDX3X***	**41203568**	**A**	**AAC**	**frameshift insertion**	**p.314ins**	**36**
**X**	***MED12***	**70356335**	**C**	**G**	**nonsynonymous SNV**	**p.L1744V**	**39**
**X**	***ZMYM3***	**70468137**	**C**	**T**	**nonsynonymous SNV**	**p.R617H**	**60**
**X**	***STAG2***	**123200027**	**C**	**A**	**nonsynonymous SNV**	**p.A700D**	**5**

Abbreviations: AA, amino acid; SNV, single nucleotide variant

**Fig 2:**
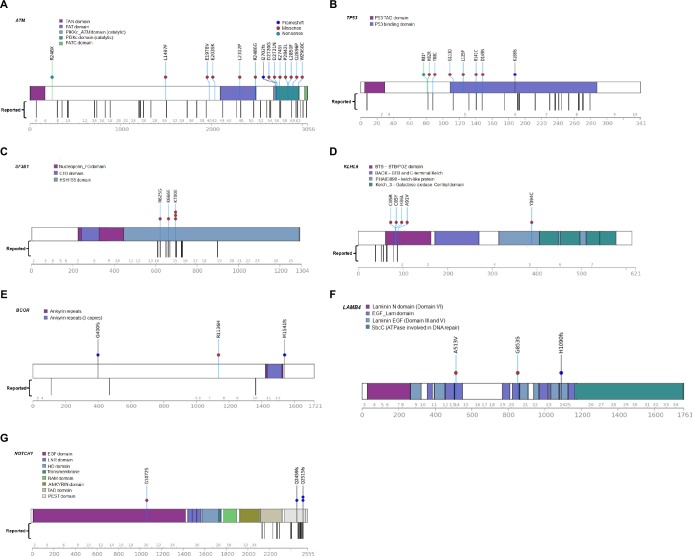
Diagrams for mutations observed in more than 5% of the cases. Genes include (A) *ATM*, (B) *TP53*, (C) *SF3B1*, (D) *KLHL6*, (E) *BCOR*, (F) *LAMB4*, and (G) *NOTCH1* (Transcript ID: ATM, NM_000051; TP53, NM_001126114; SF3B1, NM_024582; KLHL6, NM_130446; BCOR, NM_001123383; LAMB4, NM_007356; NOTCH1, NM_017617).

**Fig 3:**
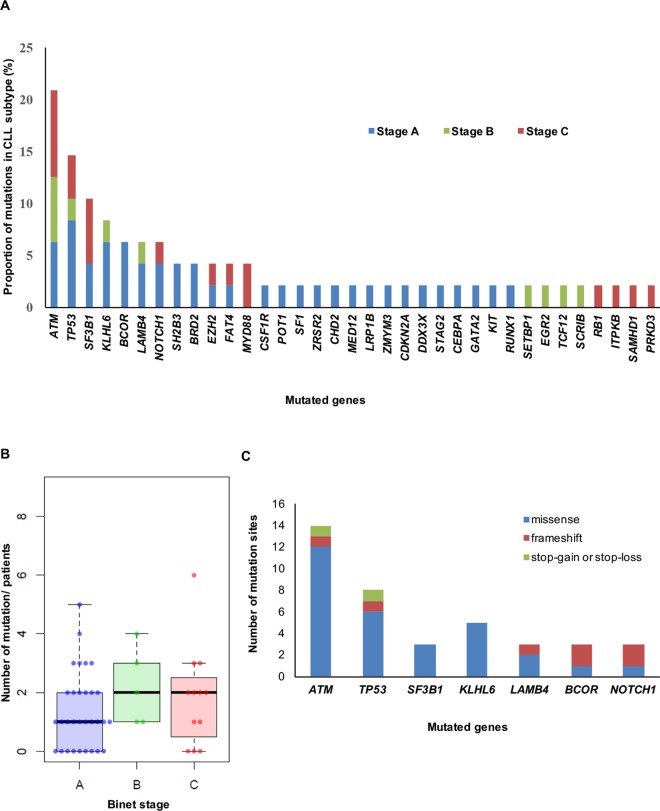
Genomic landscape of the Korean patients. (A) Frequency of gene mutations broken down by CLL stage. (B) Number of mutations according to the Binet stage. (C) Number of mutation sites in each gene that occur in more than 5% of the population.

Importantly, the mutations with high frequency over 10% were *ATM* (20.8%), *TP53* (14.6%), and *SF3B1* (10.4%). Additionally, mutations in *ATM*, *TP53*, *SF3B1*, *KLHL6*, *BCOR*, *LAMB4*, and *NOTCH1* were detected in more than 5% of the patients (in 20.8%, 14.6%, 10.4%, 8.3%, 6.25%, 6.25%, and 6.25% of the patients, respectively) (Fig 2). Missense mutation was the most frequent, followed in order by frameshift mutation and stop-gain, or stop-loss mutation. We examined these mutations (*ATM*, *TP53*, *SF3B1*, *KLHL6*, *BCOR*, *LAMB4*, and *NOTCH1*) according to Binet stage. *ATM* (n = 4) and *SF3B1* (n = 3) were the most frequent mutations in patients in Binet stage C, and *TP53* (n = 4) was the most frequent in patients in Binet stage A. The mean number of mutations according to Binet staging was 1.4 (43 mutations in 31 patients) for stage A, 2.2 (11 mutations in 5 patients) for stage B, and 1.8 (22 mutations in 12 patients) for stage C, revealing no correlation between the Binet stage and number of mutations (Fig 3).

**Fig 4:**
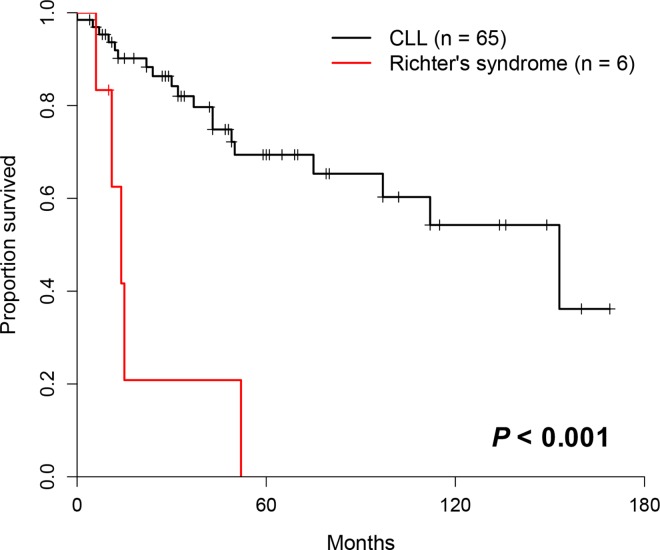
Kaplan-Meier survival curves of patients who transformed to Richter’s syndrome.

Eight percent (6/71) of the patients transformed to Richter’s syndrome and an average of 1.8 mutations were detected per one patient with Richter’s syndrome (range, 1–3). 10 different gene mutations were identified (*ZMYM3*, *CDKN2A*, *ATM*, *TP53*, *NOTCH1*, *SF3B1*, *SAMD1*, *MYD88*, *DDX3X* and *RUNX1*). The frequency of *TP53* mutation in patients with Richter’s syndrome (33.3%, 2/6) was higher than that among other CLL patients without Richter’s syndrome (11.9%, 5/42). Patients who transformed to Richter’s syndrome had a significantly shorter survival compare to the other (*P*<0.001, Fig 4).

Fifteen genes (17 indel) had at least one insertion or deletion. Known mutations includes *NOTCH1* (p.R2515Rfs*4), *TP53* (H82R, Y88C, G113D, R141C and D149N), EGR2 (E356K), *MYD88* (X160R), *ATM* (K2749I) and *SF3B1* (K666E and K700E). Among substitutions, transitions (68.5%) were more prevalent than transversions (31.5%). The most frequently mutated targets were involved in transcription (33.3% of cases) and signaling (33.3%), followed by DNA repair (20.8%), splicing (14.6%), cell cycle (14.6%), receptor/kinase (10.4%), other functions (6.25%), chromatin modification (6.25%) and cohesin (2.08%).

### Correlation between gene mutations and cytogenetics

**Fig 5:**
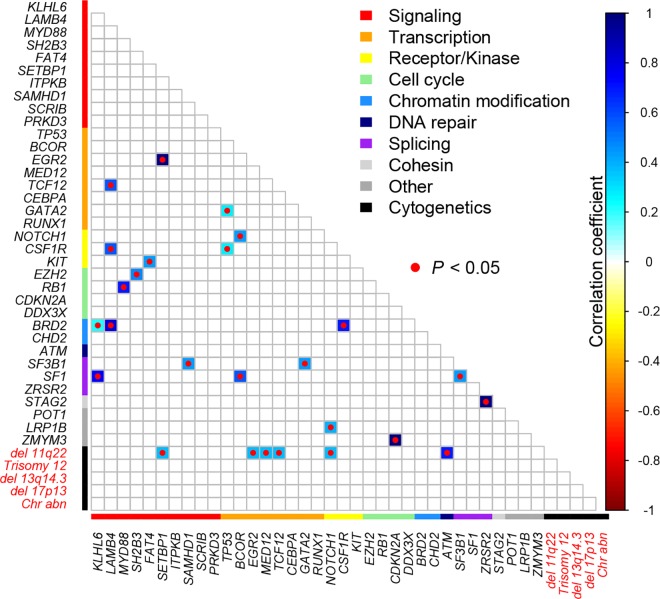
Correlation between various gene mutations and between gene mutation and cytogenetics. Correlation coefficients with raw P<0.05 were presented as colored boxes. Applying FDR correction for multiple comparison, there were no statistically significant correaltions.

The correlations between the occurrence of various mutated genes and between gene mutations and cytogenetics are shown in Fig 5. Strong positive correlations were observed for the following 8 gene-gene or gene-cytogenetics combinations: *EGR2* and *SETBP1* (R = 1), *TCF12* and *LAMB4* (R = 0.565), *GATA2* and *TP53* (R = 0.286), *NOTCH1* and *BCOR* (R = 0.438), *CSF1R* and *LAMB4* (R = 0.565), *CSF1R* and *TP53* (R = 0.286), *KIT* and *FAT4* (R = 0.432), *EZH2* and *SH2B3* (R = 0.478), *RB1* and *MYD88* (R = 0.700), *BRD2* and *KLHL6* (R = 0.225), *BRD2* and *LAMB4* (R = 0.808), *BRD2* and *CSF1R* (R = 0.700), *SF3B1* and *SAMHD1* (R = 0.428), *SF3B1* and *GATA2* (R = 0.428), *SF1* and *KLHL6* (R = 0.753), *SF1* and *BCOR* (R = 0.565), *SF1* and *SF3B1* (R = 0.428), *STAG2* and *ZRS*R2 (R = 1), *LRP1B* and *NOTCH1* (R = 0.389), *ZMYM3* and *CDKN2A* (R = 1), and 11q22 deletion and *SETBP1* (R = 0.377), 11q22 deletion and *EGR2* (R = 0.377), 11q22 deletion and *MED12* (R = 0.377), 11q22 deletion and *TCF12* (R = 0.377), 11q22 deletion and *NOTCH1* (R = 0.377), 11q22 deletion and *ATM* (R = 0.696) (raw *P*<0.05, Pearson’s correlation analysis test). While after multiple comparison by using FDR, there were no statistically significant correlations.

### Prognostic relevance of cytogenetic abnormalities and ZAP-70

**Fig 6:**
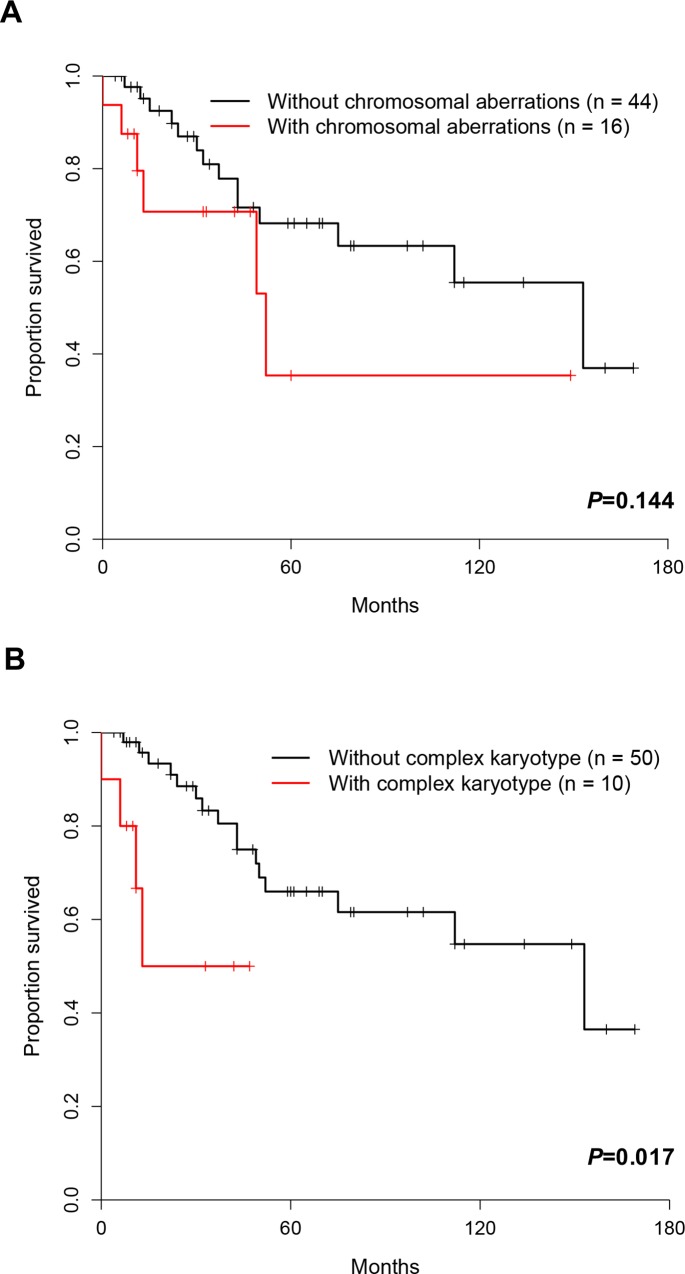
Kaplan-Meier survival curves according to cytogenetic abnormality.

**Fig 7:**
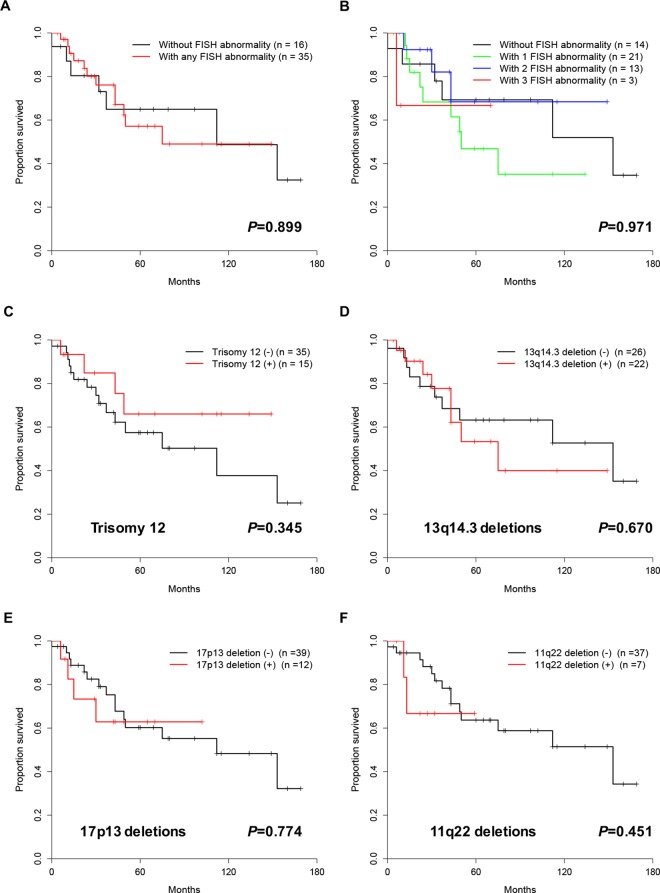
Kaplan-Meier survival curves according to abnormalities detected by FISH.

Although there was no correlation between aberrant karyotype and survival (*P* = 0.144), complex karyotypes were found to be significantly associated with poor prognosis (*P* = 0.017) (Fig 6). We also examined the relationship between FISH results and overall survival (Fig 7). It turned out that neither abnormal nor normal FISH results were correlated with overall survival (*P* = 0.899). Additionally, trisomy 12, 13q14, 17p13, and 11q23 deletion were not correlated with survival as well (*P* = 0.345, *P* = 0.670, *P* = 0.774, and *P* = 0.451, respectively). There was no correlation between prognosis and the detection of one, two or three abnormalities by FISH (*P* = 0.971). Finally, patients with the expression of ZAP-70 showed poor OS compared to patients without ZAP-70 expression (*P* = 0.007).

### Prognostic relevance of gene mutations

**Fig 8:**
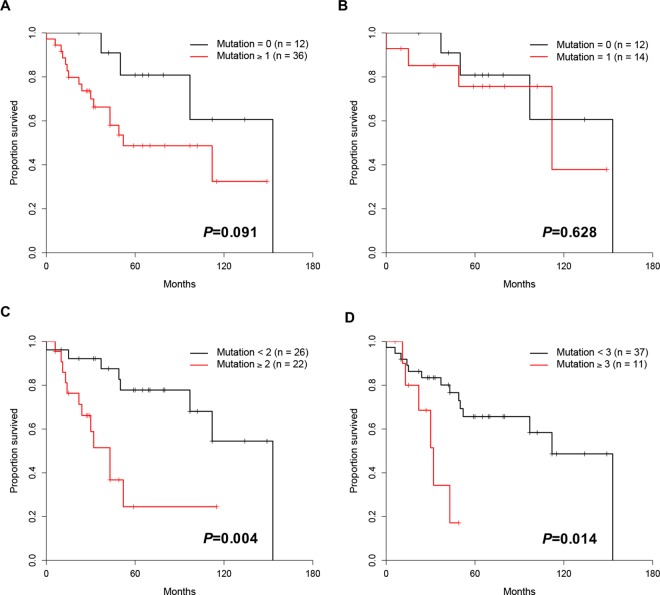
Kaplan-Meier survival curves according to the number (n< 3, n≥3) of somatic mutations.

No significant difference in survival between patients without mutation and those with mutations (*P* = 0.091). Also, there was no significant difference in survival between patients with no mutation and those with 1 mutation (*P* = 0.628), but significance difference was observed between patients with 0–1 mutation and those with ≥2 mutations (*P* = 0.004). Patients with ≥3 mutations also showed a significantly shorter survival compared to those with 0–2 mutations (*P* = 0.014) (Fig 8).

**Fig 9:**
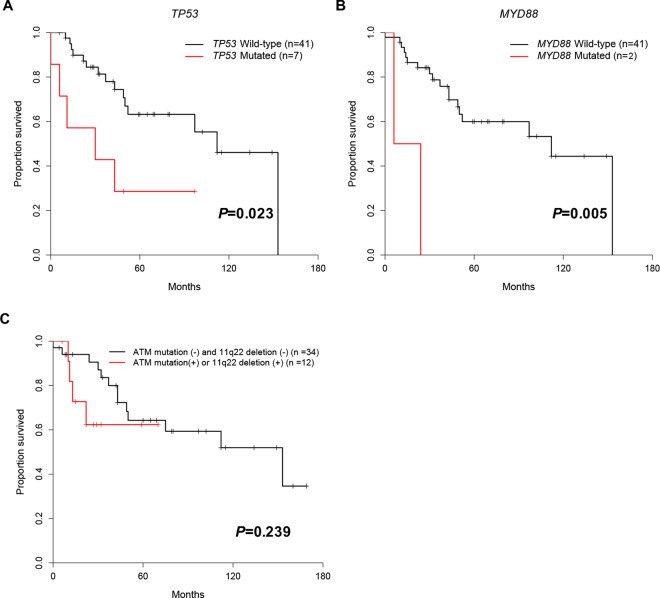
Kaplan-Meier survival curves in patients with somatic mutations. (A) *TP53*, (B) *MYD88*, and (C) *ATM* gene mutation or 11q22 deletion in Korean CLL. After applying multiple comparison by using FDR, only *MYD88* showed a tendency for adverse prognosis (P = 0.055).

**Fig 10:**
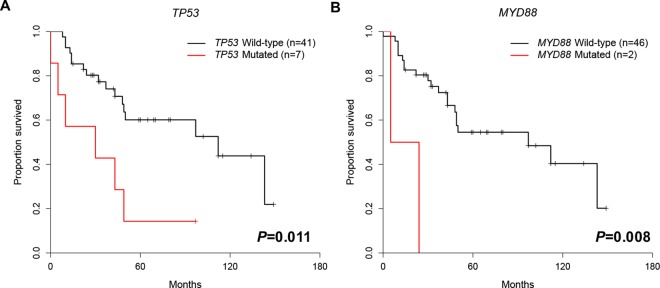
Disease-free survival curves for patients with mutations. (A) *TP53* and (B) *MYD88* genes After applying multiple comparison by using FDR, *TP53* and *MYD88* showed a tendency for shorter disease-free survival (*P* = 0.054, both).

Patients with *TP53*_*mut*_, *MYD88*_*mut*,_ *SETBP1*_*mut*_, *ITPKB*_*mut*_, *SAMHD1*_*mut*_ and *EGR2*_*mut*_ had a significantly shorter survival compared to patients without these mutations (*P* = 0.023, *P* = 0.005, *P* = 0.032, *P* = 0.011, *P* = 0.049 and *P* = 0.032, respectively) (Fig 9). However, number of patient with *SETBP1*_*mut*_, *ITPKB*_*mut*_, *SAMHD1*_*mut*_ and *EGR2*_*mut*_ was only one, of which significance is inconclusive. Additionally, applying multiple comparison by using FDR, only *MYD88*_*mut*_ showed moderate adverse prognosis (*P* = 0.055). *TP53*_*mut*_ and *MYD88*_*mut*_ were also associated with shorter disease-free survival (*P* = 0.011 and *P* = 0.008, respectively) (Fig 10). The statistical results showed that *ITPKB*_*mut*_, *TCF12*_*mut*_, and *RUNX1*_*mut*_ were also related to lower disease-free survival (*P* = 0.030, *P* = 0.010 and *P* = 0.030, respectively), but only one patient (n = 1) harbored these mutated genes, and we therefore do not draw any conclusions for these three mutated genes. However, applying multiple comparison by using FDR, *TP53*_*mut*_ and *MYD88*_*mut*_ *were* associated with shorter disease-free survival with moderate difference (*P* = 0.054, both). Mutations in the *ATM*, *SF3B1*, and *NOTCH1* genes, which are well known poor prognostic genes, were not associated with poor survival in the present study of Korean CLL patients. In contrast to the adverse prognostic impact of *ATM* gene, *ATM* mutation or *ATM* deletion was not associated with poor prognosis (*P* = 0.239, Fig 9C)

**Fig 11:**
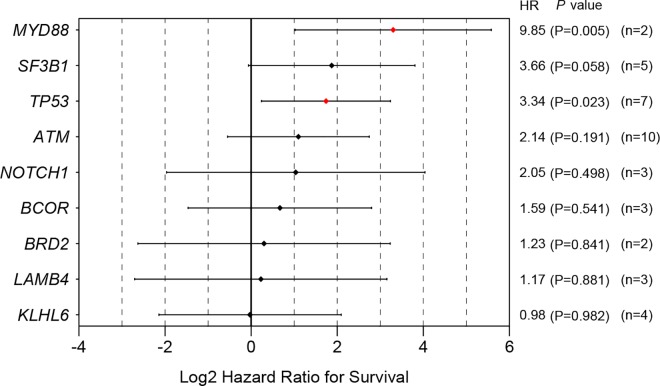
Hazard ratios with 95% confidence intervals for overall survival for each gene variant. Data of gene mutations which are shown only in one patient are not shown. Raw *P*-values are shown in this figure. After applying FDR for multiple comparison, *MYD88* mutation is the only statistically significant gene which had high hazard ratio (*P* = 0.045).

We also calculated the log hazard ratios for the overall survival in each gene variant using the conventional Cox regression model and found several that had significantly high hazard ratios: *ITPKB* (HR = 22.82, *P* = 0.011), *SF3B1* (HR = 10.98, *P* = 0.032), *EGR2* (HR = 10.98, *P* = 0.032), *MYD88* (HR = 9.85, *P* = 0.005), *SAMHD1* (HR = 8.64, *P* = 0.049) and *TP53* (HR = 3.34, *P* = 0.023) (Fig 11). Among these genes, note that *TP53* turned out to be a poor prognostic marker, as its mutation occurred in seven patients. The other genes except *TP53* (n = 7) and *MYD88* (n = 2), however, mutated in only one patient each (n = 1), which calls for a careful interpretation. However, after applying multiple comparison, *MYD88* is the only statistically significant gene which had high hazard ratio (*P* = 0.045)

## Discussion

An important contribution of this study is that it reveals that the mutation profiles of Caucasian and Korean patients differ. Although the cytogenetic profiles of CLL patients was similar between Caucasians and Koreans, the molecular genetic profile was clearly different between the two groups. Among Korean patients with CLL, cytogenetic aberrations were found in 30.4% of the patients using G-banding, and in 66.7% of the patients using FISH. These figures are similar to those of Caucasians and other Asian patients examined in previous studies [29–31]

**Fig 12:**
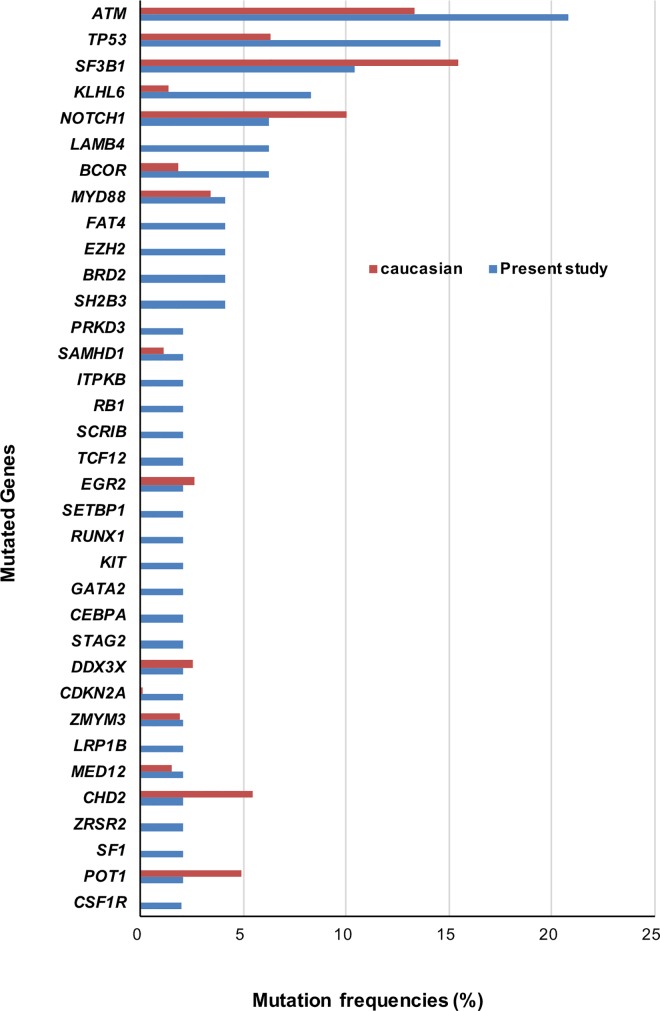
Comparison of mutation frequencies (%) between Caucasians and Koreans. Frequencies of mutation in Caucasian were calculated, based on the data of Landau et al. (2015) and Puente et al.(2015).

Meanwhile, the frequencies and patterns of mutated genes were different between Caucasians and Koreans. The most frequent mutated gene among Koreans was *ATM* (20.8%), followed in order by *TP53* (14.6%) and *SF3B1* (10.4%) (S6 Table). This result contrasts with that of Caucasians, in which around 10% of the CLL population harbor *ATM*_*mut*_ [8, 9]. Moreover, the frequency of *TP53*_*mut*_, is 2-fold higher in Koreans, while *SF3B1*_*mut*_, which is the most frequent mutation in Caucasians (21%) [8], occurs in only 10.4% of the Korean patients (Fig 12). *NOTCH1*_*mut*,_, *CHD2*_*mut*,_ and *POT1*_*mut*_ were more frequent in Caucasian CLL [8, 9] than in Korean CLL. Conversely, *KLHL6*_*mut*_ and *BCOR*_*mut*_ were more common in Koreans, while *MYD88*_*mut*_, *SAMHD1*_*mut*,_ *EGR2*_*mut*_, *DDX3X*_*mut*_, *ZMYM3*_*mut*_, and *MED12*_*mut*,_ showed similar frequencies in Koreans and Caucasians [8, 9]. Statistically, *TP53*_*mut*_ and *KLHL6*_*mut*_ were significantly more frequent in Koreans (*P* = 0.037 and *P* = 0.008), though after applying multiple comparison, there were no statistical difference in mutation frequency between Caucasians and Koreans (S7 Table). In summary, mutation frequencies in *ATM*, *TP53*, *KLHL6*, *BCOR*, and *CDKN2A* tend to be higher in Koreans than in Caucasians, while mutation frequencies in *SF3B1*, *NOTCH1*, *CHD2* and *POT1* tend to be higher in Caucasians.

The mutation profile of Korean CLL patients was different from that of Chinese patients as well. Although the mutation rates of *TP53* and *NOTCH1* found in our study were similar to those found in a Chinese study reporting mutation patterns in CLL patients [10] (15% vs. 15% and 8% vs. 6%), mutations in *SF3B1* were twice as common in Koreans (5% vs. 10%) and mutations in *MYD88* were half as common in Koreans (8% vs. 4%). Thus, we conclude that differences in the distribution of mutations were observed between Asian populations.

With regards to poor prognostic markers, the poor prognostic markers of Korean patients identified in this study are complex karyotypes, *TP53* mutation, and *MYD88* mutation. Among these genes, *TP53* mutation has already been reported as a poor prognostic marker in studies on both Caucasian [11] and Chinese CLL patients [10]. On the other hand, *MYD88* mutation has not been discussed as a poor prognostic marker in previous studies on Caucasian patients, but it has not been studied with Chinese patients. We assume that the relationship between *MYD88* and poor prognosis is a unique characteristic of Korean patients, in contrast with Caucasian patients. Ethnic differences might underlie this discrepancy. Studies of Caucasian populations have reported that mutations in *TP53*, *BIRC3*, *ATM*, *NOTCH1*, and *SF3B1* are associated with poor prognosis [11]. In this study, mutations in *TP53*, *MYD88*, *SETBP1*, *ITPKB*, *SAMHD1*, and *EGR2* were associated with poor prognosis, but the number of patient with *SETBP1*_*mut*_, *ITPKB*_*mut*_, *SAMHD1*_*mut*_ and *EGR2*_*mut*_ that had these mutations was only one, of which prognostic significance might be inconclusive. Only *TP53* mutation had similar adverse prognostic associations both in our study population and in Caucasians. Compared to mutations in *TP53* and *NOTCH1* that were identified as poor prognostic factors in the Chinese study [10], *TP53* commonly showed a poor prognostic association among Caucasians, Chinese and Koreans.

This study found several genes that were reported only in Korean CLL patients and not in Caucasians. In the present study, sixty sites of novel mutation which were not found in Caucasian CLL were identified. These novel mutations are in the *LAMB4*, *SH2B3*, *RUNX1*, *SCRIB*, *KIT*, *GATA2*, *CEBPA*, *TCF12*, *STAG2*, *ZRSR2*, *SF1*, *CSF1R*, and *SETBP1* genes. A *LAMB4* mutation has been reported in MDS [32]; this protein-coding gene mediates the attachment and migration of cells into tissues during embryonic development [33, 34]. *RUNX*, *ZRSR2*, and *SF1* have also been reported in MDS [35, 36]; *RUNX1* plays an important role in regulating the transcription of many tumor-suppressor genes [37], while *ZRSR2* is an essential component of the splicing machinery [36], and SF1 is a component of the RNA-splicing machinery [36]. *GATA2*, *CEBPA* and *CSF1R* have been reported in MDS and acute myeloid leukemia (AML) [38–40]. *GATA2* is responsible for the proliferation and survival of early hematopoietic cells [41], and *CEBPA* is a transcription factor that plays important roles in myeloid differentiation [42]. *CSF1R* instructs myeloid lineage fate decisions in hematopoietic stem cells [43]. *SH2B3* (regulates integrin signaling in endothelial cells [44]) and *TCF12* (control of lymphoid differentiation [45]) have been linked to acute lymphocytic leukemia [45, 46]. *KIT* encodes a transmembrane glycoprotein [47] that has been associated with AML [48]. *SCRIB* has been reported in myeloproliferative neoplasms and regulates the differentiation of planar cell polarity [49]. *STAG2* has been reported in myeloid diseases, such as MDS, AML and chronic myelomonocytic leukemia, and encodes components of the cohesion complex [50]. *SETBP1* mutations are observed in atypical chronic myeloid leukemia [51]. Massively parallel sequencing provides new insights that enable the systematic discovery of the genetic aberrations underlying diseases and can lead to the identification of new druggable targets. Even with the major breakthroughs achieved through NGS studies, recurrent mutations discovered by NGS need to be investigated with subsequent functional study associated with CLL.

Binet staging is one of the clinical staging systems that is commonly used to predict prognosis. In the present study, there was no correlation between Binet stage and the number of somatic mutations, and mutations in adverse prognostic genes such as *TP53* and *MYD88* was not found more frequently in stage C, compared to other stages (*P =* 0.140). Furthermore, allele burdens of mutated genes did not correlate with Binet stages. These results might result from small number of enrolled patients in Korea. On the other hand, gene mutations can be used independently in predicting prognosis in Korean CLL.

With regards to Richter’s syndrome, patients with Richter’s syndrome were reported to harbor more mutated genes up to 20 genes than patients with CLL (an average of 1.8 mutations per case) [52]. *TP53* disruption, *c-MYC* abnormalities [53], *NOTCH1* mutation [54], *BRAF* [55], and *CDKN2A/B* mutation [52] were reported to be found in Richter’s syndrome. In the present study, frequency of Richter’s syndrome was 8% (6/71 patients) in Korean CLL. *TP53*, *NOTCH1*, and *CDKN2A* mutations were identified in patients with Richter’s syndrome cases, but mutation frequencies of these genes did not differ between patients with Richter’s syndrome and patients without Richter’s syndrome (*P =* 0.206, *P =* 0.336, and *P =* 0.125, respectively).

For the cytogenetic aberrations, the frequencies of cytogenetic aberrations were somewhat similar to those reported in previous studies in Caucasians: 13q14 deletion was the most frequent chromosomal aberration, followed by trisomy 12, 17p deletion and 11q deletion (S8 Table). In a study in Korean patients (n = 48), trisomy 12 was the most frequently observed abnormality [56], while another Korean study (n = 16) [30] reported rankings similar to those reported here. In Caucasian CLL studies, trisomy 12 is associated with advanced disease and a less favorable prognosis [57], while CLL patients with 11q deletion or 17p deletion had poorer outcomes, compared with patients with normal karyotypes [58]. However, in the present study, neither the 11q nor 17p deletion showed poor outcomes, which is probably due to the low number of patients.

In conclusion, this study is the first comprehensive NGS-based study of Korean patients, although a limited number of cases (n = 48) were analyzed. Major limitation of the present study is an absence of germline DNA of patients with CLL. To overcome the absence of germline DNA, we performed targeted sequencing in 276 healthy control persons in Korea and filtered all the variants which were present in normal control specimen.

The results of our study contribute to the characterization of the mutational landscape of the Asian population. The gene mutations of Caucasians and Asians turned out to be different, which leads to the conclusion that there is an ethnic difference. The prognostic genetic markers of Korean CLL patients were also different from those of Caucasians. The novel genes discovered in the present study can possibly be strong druggable targets for the treatment of CLL. The true etiology of ethnic difference in CLL is still unknown; thus, studies including clinical variables based on well-defined CLL cohorts in Asian would be future area of interest.

## Supporting Information

S1 Table Gene panel for targeted sequencing. (DOCX)

S2 Table Primer sequences used for Sanger sequencing of 16 selected variants. (DOCX)

S3 Table Baseline clinical features of patients with CLL. (DOCX)

S4 Table Cytogenetic aberrations detected by G-banding and FISH studies in 60 Korean Patients with CLL. (DOCX)

S5 Table Summary of target-based sequencing results. (DOCX)

S6 Table Frequencies of mutated genes (%) in Caucasian, Chinese and Korean populations. (DOCX)

S7 Table Mutation percentage difference between Caucasians and Koreans. (DOCX)

S8 Table Incidence (%) of chromosomal abnormalities in patients with CLL (previous and present studies). (DOCX)
