# Supplementary material for: Genomic Profile of Chronic Lymphocytic Leukemia in Korea Identified by Targeted Sequencing
Source: PLoS One. 2016 Dec 13;11(12):e0167641. doi: 10.1371/journal.pone.0167641 (PMC5154520; doi:10.1371/journal.pone.0167641)
Supplement: S1 Table — (DOCX) [file pone.0167641.s001.docx]

**S1 Table. Gene panel for targeted sequencing.**

| **Genes** | **NCBI Id.** | **Position** | **Pathway/Ontology** |
| --- | --- | --- | --- |
| ***ASXL1*** | **171023** | **20q11.1** | **Chromatin modification** |
| ***ATM*** | **472** | **11q22.3** | **DNA repair** |
| ***ATRX*** | **546** | **Xq21.1** | **Chromatin modification** |
| ***BARD1*** | **580** | **2q35** | **DNA repair** |
| ***BCOR*** | **54880** | **Xp11.14** | **Transcription** |
| ***BIRC3*** | **330** | **11q22.2** | **Receptor/Kinases** |
| ***BRAF*** | **673** | **7q34** | **RAS pathway** |
| ***BRCC3*** | **79184** | **Xq28** | **DNA repair** |
| ***BRD2*** | **6046** | **6p21.3** | **Transcription** |
| ***BRD4*** | **23476** | **19p13.1** | **Other** |
| ***CARD6*** | **84674** | **5p13.1** | **Other** |
| ***CBL*** | **867** | **11q23.3** | **RAS pathway** |
| ***CCND1*** | **595** | **11q13.3** | **Cell cycle** |
| ***CDKN2A*** | **1029** | **9p21** | **Cell cycle** |
| ***CEBPA*** | **1050** | **19q13.1** | **Transcription** |
| ***CHD2*** | **1106** | **15q26.1** | **Other** |
| ***CSF1R*** | **1436** | **5q32** | **Receptor/Kinases** |
| ***CSF3R*** | **1441** | **1p34.3** | **Receptor/Kinases** |
| ***DAP3*** | **7818** | **1q22** | **Other** |
| ***DDX3X*** | **1654** | **Xp11.4** | **Other** |
| ***DIS3*** | **22894** | **13q22.1** | **Other** |
| ***DNMT3A*** | **1788** | **2p23** | **DNA methylation** |
| ***EEF1E1*** | **9521** | **6p24.3** | **Other** |
| ***EGR2*** | **1959** | **10q21.3** | **Transcription** |
| ***ETV6*** | **2120** | **12p13.2** | **Transcription** |
| ***EZH2*** | **2146** | **7q35-36** | **Chromatin modification** |
| ***FAM46C*** | **54855** | **1p12** | **Other** |
| ***FAT4*** | **79633** | **4q28.1** | **Other** |
| ***FBXW7*** | **55294** | **4q31.3** | **Receptor/Kinases** |
| ***FLT3*** | **2322** | **13q12** | **Receptor/Kinases** |
| ***GATA1*** | **2623** | **Xp11.23** | **Transcription** |
| ***GATA2*** | **2624** | **3q21.3** | **Transcription** |
| ***HIST1H1E*** | **3008** | **6p22.2** | **Other** |
| ***IDH1*** | **3417** | **2q33.3** | **DNA methylation** |
| ***IDH2*** | **3418** | **15q26.1** | **DNA methylation** |
| ***IKZF1*** | **10320** | **7p13** | **Transcription** |
| ***ITPKB*** | **3707** | **1q42.12** | **Signaling** |
| ***JAK2*** | **3717** | **9p24** | **Receptor/Kinases** |
| ***KIAA0355*** | **9710** | **19q13.11** | **Other** |
| ***KIT*** | **3815** | **4q12** | **Receptor/Kinases** |
| ***KLHL6*** | **89857** | **3q27.1** | **Other** |
| ***KRAS*** | **3845** | **12p12.1** | **RAS pathway** |
| ***LAMB4*** | **22798** | **7q31.1** | **Other** |
| ***LRP1B*** | **53353** | **2q21.2** | **Other** |
| ***MAPK1*** | **5594** | **22q11.22** | **Signal/Kinase** |
| ***MED12*** | **9968** | **Xq13.1** | **Other** |
| ***MPL*** | **4352** | **1p34.2** | **Receptor/Kinases** |
| ***MYD88*** | **4615** | **3p22.2** | **Signaling** |
| ***NF1*** | **4763** | **17q11.2** | **RAS pathway** |
| ***NFKBIE*** | **4794** | **6p21.1** | **Other** |
| ***NOTCH1*** | **4851** | **9q34.3** | **Receptor/Kinases** |
| ***NPM1*** | **4869** | **5q35** | **Transcription** |
| ***NRAS*** | **4893** | **1p13.2** | **RAS pathway** |
| ***PHF6*** | **84295** | **Xq26.2** | **Transcription** |
| ***PLEKHG5*** | **57449** | **1p36.31** | **Other** |
| ***POLG*** | **5428** | **15q25** | **Other** |
| ***POT1*** | **25913** | **7q31.33** | **Other** |
| ***PRKD3*** | **23683** | **2p22.2** | **Signaling** |
| ***PRPF40B*** | **25766** | **12q13.12** | **Splicing** |
| ***PTEN*** | **5728** | **10q23.3** | **Other** |
| ***PTPN11*** | **5781** | **12q24.1** | **RAS pathway** |
| ***RAD21*** | **5885** | **8q24.11** | **Cohesin** |
| ***RB1*** | **5925** | **13q14** | **Cell cycle** |
| ***RIPK1*** | **8737** | **6p25.2** | **Other** |
| ***RUNX1*** | **861** | **21q22.3** | **Transcription** |
| ***SAMHD1*** | **25939** | **20q11.23** | **Other** |
| ***SCRIB*** | **23513** | **8q24.3** | **Other** |
| ***SETBP1*** | **26040** | **18q12.3** | **Other** |
| ***SF1*** | **7536** | **11q13.1** | **Splicing** |
| ***SF3A1*** | **10291** | **22q12.2** | **Splicing** |
| ***SF3B1*** | **23451** | **2q33.1** | **Splicing** |
| ***SH2B3*** | **10019** | **12q24.12** | **Signaling** |
| ***SMARCA2*** | **6595** | **9p24.3** | **Other** |
| ***SMC1A*** | **8243** | **Xp11.22** | **Cohesin** |
| ***SMC3*** | **9126** | **10q25.2** | **Cohesin** |
| ***SRSF2*** | **6427** | **17q25.1** | **Splicing** |
| ***STAG2*** | **10735** | **Xq25** | **Cohesin** |
| ***TCF12*** | **6938** | **15q21.3** | **Transcription** |
| ***TET2*** | **54790** | **4q24** | **DNA methylation** |
| ***TGM7*** | **116179** | **15q15.2** | **Other** |
| ***TP53*** | **7157** | **17p13.1** | **Transcription** |
| ***U2AF1*** | **7307** | **21q22.3** | **Splicing** |
| ***U2AF2*** | **11338** | **19q13.42** | **Splicing** |
| ***WT1*** | **7490** | **11p13** | **Transcription** |
| ***XPO1*** | **7514** | **2p15** | **Other** |
| ***ZMYM3*** | **9203** | **Xq13.1** | **Other** |
| ***ZRSR2*** | **8233** | **Xp22.1** | **Splicing** |
