# Supplementary material for: Genomic Profile of Chronic Lymphocytic Leukemia in Korea Identified by Targeted Sequencing
Source: PLoS One. 2016 Dec 13;11(12):e0167641. doi: 10.1371/journal.pone.0167641 (PMC5154520; doi:10.1371/journal.pone.0167641)
Supplement: S2 Table — (DOCX) [file pone.0167641.s002.docx]

**S2 Table. Primer sequences used for Sanger sequencing of 16 selected variants**

| **Chr** | **Gene** | **Position** | **Reference** | **Alternative** | **Forward (5'→3')** | **Reverse (5'→3')** |
| --- | --- | --- | --- | --- | --- | --- |
| **2** | ***SF3B1*** | **198267484** | **G** | **C** | **AAGAATAGCTATCTGTTGTACA** | **TGTTTATGGAATTGATTATGGAAA** |
| **7** | ***LAMB4*** | **107706935** | **C** | **T** | **AAAATTAAAGTGCATATGAATTCC** | **GCTCCAGCATACTTCTCTTT** |
| **7** | ***EZH2*** | **148514402** | **CAGCACCACTCCACTCCACATTCTCAG** | **-** | **CCTGTCTACATGTTTTGGTCCC** | **ACGATGGGTTAGTGTTTTGCC** |
| **11** | ***ATM*** | **108183152** | **A** | **T** | **TAAGTGATTTATTCTGTTTTGTTTG** | **CTGTACAGTGTCTATAACAAAATAA** |
| **11** | ***ATM*** | **108186757** | **G** | **A** | **GTGGAGGGAAGATGTTACAA** | **CCAACATACTGAAATAACCTCA** |
| **11** | ***ATM*** | **108201089** | **C** | **G** | **GATACACAGTAAAGGTTCAGC** | **TACAAAGAGGTATACACGATTC** |
| **11** | ***ATM*** | **108206579** | **A** | **G** | **AGGTATTTAATTATTTGGGAGACT** | **TTATATGTTTTTGGTGAACTAACA** |
| **11** | ***ATM*** | **108206581** | **G** | **A** | **GTATTTAATTATTTGGGAGACTGT** | **TTTTATATGTTTTTGGTGAACTAAC** |
| **11** | ***ATM*** | **108206666** | **A** | **T** | **ATAAACTGTACTTGTTTATTCATGC** | **GCCTCCCAAAGCATTATGAA** |
| **11** | ***ATM*** | **108216576** | **C** | **T** | **TATATTCTCTATTTAAAGGAGGTGC** | **ACTCAGAATGTAGAAAAAGTGC** |
| **11** | ***ATM*** | **108224508** | **A** | **C** | **AACTACTGTACATACTAGTGTTC** | **ATTTTGACATCAAAAATTATTTCCC** |
| **11** | ***ATM*** | **108235838** | **G** | **T** | **CCCCATCAACTACCATGTGA** | **ATCTGAAAAACTGACAACAGG** |
| **17** | ***TP53*** | **7577121** | **G** | **A** | **CTTCTTGTCCTGCTTGCTTA** | **TAGGCTCCAGAAAGGACAAG** |
| **17** | ***TP53*** | **7578190** | **T** | **C** | **ATTTACTTTGCACATCTCATG** | **GCCTCTGATTCCTCACTGAT** |
| **17** | ***TP53*** | **7578208** | **T** | **C** | **ATGGGGTTATAGGGAGGTCA** | **GCCTCTGATTCCTCACTGAT** |
| **17** | ***TP53*** | **7578212** | **G** | **A** | **GGTTATAGGGAGGTCAAATAAG** | **GCCTCTGATTCCTCACTGAT** |

**Abbreviations: chr, chromosome**
