# Supplementary material for: Genomic Profile of Chronic Lymphocytic Leukemia in Korea Identified by Targeted Sequencing
Source: PLoS One. 2016 Dec 13;11(12):e0167641. doi: 10.1371/journal.pone.0167641 (PMC5154520; doi:10.1371/journal.pone.0167641)
Supplement: S3 Table — (DOCX) [file pone.0167641.s003.docx]

**S3 Table. Baseline clinical features of patients with CLL.**

| **Parameter** | **Patient numbers**  **(ratios, ranges or percentages)** |
| --- | --- |
| **Demographic characteristics** | **71** |
| **Median age (years) at diagnosis** | **61 (23-81)** |
| **<65** | **44 (62.0%)** |
| **≥65** | **27 (38.0%)** |
| **Sex** |  |
| **Male** | **45 (63.4%)** |
| **Female** | **26 (36.6%)** |
|  |  |
| **Clinical characteristics^*^** | **71** |
| **WBC (×10^9^/L)** | **15,040 (1,340-353,050)** |
| **Lymphocytes (×10^9^/L)** | **11,248 (670-247,135)** |
| **Hb (g/L)** | **12.8 (7-17)** |
| **PLT (×10^9^/L)** | **163 (41-389)** |
| **Binet stage** |  |
| **A** | **46 (64.8%)** |
| **B** | **10 (14.1%)** |
| **C** | **15 (21.1%)** |
|  |  |
| **Cytogenetics** | **56^*^** |
| **Normal karyotype** | **39 (69.6%, 39/56)** |
| **Aberrant karyotype** | **17 (30.4%, 17/56)** |
|  |  |
| **Cases with follow-up** | **71** |
| **Median follow-up, in months** | **37** |
| **Median overall survival, in months** | **112** |
| **5-year overall survival** | **63.2%** |
| **10-year overall survival** | **49.4%** |

Abbreviations: WBC, white blood cells; Hb, hemoglobin; PLT, platelet; OS overall survival.
