# Supplementary material for: Genomic Profile of Chronic Lymphocytic Leukemia in Korea Identified by Targeted Sequencing
Source: PLoS One. 2016 Dec 13;11(12):e0167641. doi: 10.1371/journal.pone.0167641 (PMC5154520; doi:10.1371/journal.pone.0167641)
Supplement: S4 Table — (DOCX) [file pone.0167641.s004.docx]

**S4 Table. Cytogenetic aberrations detected by G-banding and FISH studies in 60 Korean Patients with CLL**

| **Patient No.** | **Age/**  **Sex** | **Binet Stage** | **Karyotype** | | **FISH** |  |  |  |
| --- | --- | --- | --- | --- | --- | --- | --- | --- |
|  |  |  |  |  | **+12 (%)** | **D13S319 (%)** | **17p13 del (%)** | **11q22**  **del (%)** |
| **1** | **54/F** | **A** | **46,XX[20]** | | **0** | **2.0** | **2.0** | **1.5** |
| **2** | **71/F** | **A** | **46,XX[20]** | | **0** | **0.5** | **3** | **0.5** |
| **3** | **60/M** | **A** | **46,XY[19]** | | **0** | **1.0** | **0.5** | **0.5** |
| **4** | **64/M** | **C** | **insufficient mitosis** | | **0** | **51.0** | **0.5** | **ND** |
| **5** | **67/M** | **A** | **46,XY[18]** | | **69.5** | **0.0** | **2** | **0** |
| **6** | **62/M** | **B** | **44,XY,,der(13;14)(q10;q10), -14[12]** | | **68.5** | **24.5** | **1.5** | **0** |
| **7** | **60/M** | **A** | **46,XY[15]** | | **0** | **27.0** | **2** | **0** |
| **8** | **79/M** | **A** | **46,XY[19]** | | **0** | **78.5** | **1.5** | **0.5** |
| **9** | **67/F** | **A** | **no mitosis** | | **0** | **4.5** | **4.5** | **1.0** |
| **10** | **38/M** | **A** | **ND** | | **0.5** | **60.0** | **6** | **55.0** |
| **11** | **66/F** | **A** | **46,XX[18]** | | **0** | **2.5** | **4.5** | **0** |
| **12** | **59/F** | **C** | **46,XX[20]** | | **8** | **0** | **0** | **3.5** |
| **13** | **66/F** | **B** | **46,XX,add(2)(q37)[4],add(3)(q29)[5],del(4)(q10)[4][cp5]/46,XX[8]** | | **0** | **0** | **0** | **1.0** |
| **14** | **43/M** | **A** | **46,XY[20]** | | **0** | **14** | **0** | **1.0** |
| **15** | **70/F** | **A** | **46,XX[14]** | | **3** | **6** | **0** | **0.5** |
| **16** | **62/M** | **A** | **46,XY[20]** | | **0** | **5** | **40** | **0.5** |
| **17** | **63/M** | **A** | **No mitosis** | | **62** | **0** | **0** | **1.0** |
| **19** | **76/M** | **C** | **ND** | | **0** | **ND** | **0** | **ND** |
| **20** | **51/M** | **A** | **46,XY[10]** | | **0** | **0** | **0** | **ND** |
| **21** | **46/M** | **B** | **46~47,XY,add(7)(p22),del(10)(p13),+mar[cp3]/46,XY[8]** | | **0** | **0** | **0** | **91.0** |
| **22** | **61/M** | **A** | **46,XY[21]** | | **0** | **0** | **0** | **0** |
| **23** | **61/M** | **A** | **46,XY[13]** | | **0** | **36** | **0** | **0** |
| **24** | **53/F** | **A** | **46,XX[21]** | | **ND** | **ND** | **ND** | **ND** |
| **26** | **53/M** | **A** | **46,XY[20]** | | **4** | **50** | **4** | **1.0** |
| **27** | **57/F** | **A** | **46,XX[20]** | | **0** | **0** | **0** | **2.5** |
| **28** | **63/F** | **A** | **46,XX[11]** | | **ND** | **ND** | **ND** | **ND** |
| **29** | **67/F** | **C** | **ND** | | **3.7** | **ND** | **0** | **ND** |
| **30** | **78/F** | **A** | **47,XX,+12[2]/46,XX[18]** | | **40.5** | **0** | **0** | **1.0** |
| **31** | **66/M** | **A** | **46,XY[6]** | | **0** | **87.0** | **0** | **89.0** |
| **32** | **36/M** | **C** | **46,XY,add(14)(q32),inc[1]/46,XY[19]** | | **0** | **0** | **0** | **1.0** |
| **33** | **79/F** | **A** | **46,XX[10]** | | **2** | **13** | **0** | **4.0** |
| **34** | **65/M** | **C** | **46,XY[20]** | | **0** | **55** | **0** | **1.0** |
| **35** | **66/M** | **A** | **ND** | | **ND** | **ND** | **91.5** | **ND** |
| **36** | **58/M** | **A** | **46,XY[21]** | | **0** | **0** | **5** | **ND** |
| **37** | **56/M** | **A** | **47,XY,-2,+3,+add(3)(p?13),der(?3;15)(q10;q10),-5,-6,del(?10)(p10),del(13)(q12q14),add(?14)(p10),-17,+3mar[3]/46,XY[25]** | | **0** | **33** | **7** | **0** |
| **38** | **55/F** | **A** | **46,XX[17]** | | **0** | **57** | **77** | **0** |
| **39** | **55/M** | **A** | | **46,XY,der(15)t(8;15)(q21.2;q26.1)[6]/46,XY[14]** | **0** | **6** | **0** | **11.5** |
| **41** | **62/F** | **C** | **48,XX,t(9;14)(p13;q32),dup(12)(q13q24.3),+15,+16[5]/47,sl,dup(1)(q?25q21),t(2;3)(p25;q21),-16[8]/47,sdl1,add(1)(p35)[3]/47,sdl1,+6,der(6;21)(p10;q10)[3]/48,sl,dup(1)(q21q32)[2]/46,XX[2]** | | **0** | **0** | **0** | **1.5** |
| **42** | **51/M** | **B** | **46,XY[20]** | | **2** | **0** | **4** | **1.5** |
| **44** | **71/F** | **C** | **46,XX,21pstk+[20]** | | **0** | **0** | **0** | **ND** |
| **45** | **81/F** | **C** | **46,XX[20]** | | **0** | **49** | **0** | **ND** |
| **46** | **55/F** | **A** | **46,XX[20]** | | **0** | **35** | **0** | **3.5** |
| **47** | **70/M** | **A** | **46,XY[5]** | | **7** | **9** | **5** | **0.5** |
| **49** | **46/F** | **A** | **47,XX,+12[1]/46,XX[28]** | | **37.5** | **0** | **0** | **0** |
| **50** | **55/M** | **A** | **46,XY[20]** | | **0** | **56** | **0** | **1.0** |
| **51** | **68/F** | **A** | **46~47,XX,add(1)(p32),add(2)(p10),add(7)(q?32),add(17)(p10),add(19)(q13.3),+mar,inc[2]/46,XX[18]** | | **ND** | **ND** | **ND** | **ND** |
| **52** | **72/F** | **A** | **52~56,X,+add(X)(q22),del(X)(q26),+der(1;16)(q10;p10),+3,+3,**  **del(4)(q31),+add(6)(q23),+del(6)(q15),add(6)(q23)x2,+7,**  **+del(7)(q22),+del(11)(q23),+13,del(15)(q15q25),+21,-22,+mar,inc[2]/46,XX[4]** | | **ND** | **ND** | **ND** | **ND** |
| **54** | **47/M** | **B** | **46,XY[20]** | | **ND** | **ND** | **ND** | **ND** |
| **55** | **49/F** | **A** | **46,XX[20]** | | **ND** | **ND** | **ND** | **ND** |
| **56** | **43/M** | **A** | **46,XY[20]** | | **ND** | **ND** | **ND** | **ND** |
| **57** | **54/M** | **B** | **46,XY[30]** | | **0** | **0** | **1.5** | **36.0** |
| **58** | **74/M** | **B** | **46,XY[20]** | | **ND** | **ND** | **ND** | **ND** |
| **59** | **49/M** | **A** | **46,XY[20]** | | **0** | **3** | **6** | **1.0** |
| **60** | **49/M** | **A** | **47,XY+12[2]/46,XY[18]** | | **ND** | **ND** | **ND** | **ND** |
| **61** | **66/F** | **A** | **No mitosis** | | **5** | **0** | **3** | **2.0** |
| **62** | **66/M** | **C** | **46,X,-Y,der(1)add(1)(p22)add(1)(q?42),add(2)(q?23),add(3)(p25),+4,-13,add(14)(p10),add(17)(p11.2),+mar[12]/46~48,idem, add(6)(q21),del(6)(q?23q25),i(8)(q10),+14,-add(14),+1~2mar,inc[cp8]** | | **6** | **59** | **61** | **2.0** |
| **63** | **55/M** | **C** | **38~46,XY,del(1)(p34),del(2)(p21),add(3)(q21),add(6)(p23),del(11)((q?24),add(16)(q22),del(17)(p12),+mar[cp5]/46,XY[9]** | | **0** | **0** | **29** | **66.0** |
| **64** | **46/F** | **C** | **46,XX[20]** | | **0** | **26** | **0** | **12.0** |
| **65** | **63/F** | **A** | **46,XX[20]** | | **1** | **3.5** | **0** | **5.0** |
| **66** | **46/M** | **A** | **46,XY[30]** | | **ND** | **ND** | **ND** | **ND** |
| **67** | **23/F** | **B** | **46,XX,i(8)(q10),del(11)(q13q23),add(18)(p11.3)[cp8]/46,XX[12]** | | **ND** | **ND** | **ND** | **ND** |
| **69** | **67/M** | **B** | **46,XY[20]** | | **ND** | **ND** | **ND** | **ND** |
| **70** | **71/M** | **C** | **48,XY,+3,+12,t(14;19)(q32;q13.3)[9]/46,XY[6]** | | **70** | **0** | **0** | **1.0** |
| **71** | **46/M** | **B** | **46,XY[20]** | | **ND** | **ND** | **ND** | **ND** |

ND; not determined
