# Supplementary material for: Genomic Profile of Chronic Lymphocytic Leukemia in Korea Identified by Targeted Sequencing
Source: PLoS One. 2016 Dec 13;11(12):e0167641. doi: 10.1371/journal.pone.0167641 (PMC5154520; doi:10.1371/journal.pone.0167641)
Supplement: S5 Table — (DOCX) [file pone.0167641.s005.docx]

**S5 Table. Summary of target-based sequencing results**

| **Variable** | **Finding** |
| --- | --- |
| **Total no. of reads** | **6,644,998** |
| **Reads mapped to the target region, %** | **98.9** |
| **Target coverage per base (mean)** | **231 X** |
| **Target base pairs covered ≥10 X, %** | **97.5** |
| **Target base pairs covered ≥100 X, %** | **85.4** |
| **Total % of variants discovered** |  |
| **Missense** | **69.0 (49/71)** |
| **Frameshift** | **18.3 (13/71)** |
| **Stop- gain or stop-loss** | **7.0 (5/71)** |
| **Non-frameshift** | **5.6 (4/71)** |
