# Supplementary material for: Genomic Profile of Chronic Lymphocytic Leukemia in Korea Identified by Targeted Sequencing
Source: PLoS One. 2016 Dec 13;11(12):e0167641. doi: 10.1371/journal.pone.0167641 (PMC5154520; doi:10.1371/journal.pone.0167641)
Supplement: S6 Table — (DOCX) [file pone.0167641.s006.docx]

**S6 Table. Frequencies of mutated genes (%) in Caucasian, Chinese and Korean populations**

| **Gene** | **Landau et al. [1] ,**  **USA (n=538)** | **Puente et al [2], Spain (n=452)** | **Mean of Caucasian studies^*^**  **(n=990)** | **Xia et al. [3], China ^†^ (n=307)** | **present study,**  **Korea (n=48)** |
| --- | --- | --- | --- | --- | --- |
| ***ATM*** | **15%** | **11%** | **13%** |  | **21%** |
| ***TP53*** | **7%** | **5%** | **6%** | **15%** | **15%** |
| ***SF3B1*** | **21%** | **9%** | **15%** | **5%** | **10%** |
| ***KLHL6*** |  | **3%** | **1%** |  | **8%** |
| ***BCOR*** | **2%** | **1%** | **2%** |  | **6%** |
| ***LAMB4*** |  |  |  |  | **6%** |
| ***NOTCH1*** | **8%** | **13%** | **10%** | **8%** | **6%** |
| ***SH2B3*** |  |  |  |  | **4%** |
| ***BRD2*** |  |  |  |  | **4%** |
| ***EZH2*** |  |  |  |  | **4%** |
| ***FAT4*** |  |  |  |  | **4%** |
| ***MYD88*** | **3%** | **4%** | **3%** | **8%** | **4%** |
| ***CSF1R*** |  |  |  |  | **2%** |
| ***POT1*** | **7%** | **3%** | **5%** |  | **2%** |
| ***SF1*** |  |  |  |  | **2%** |
| ***ZRSR2*** |  |  |  |  | **2%** |
| ***CHD2*** | **5%** | **6%** | **5%** |  | **2%** |
| ***MED12*** | **2%** | **1%** | **2%** |  | **2%** |
| ***LRP1B*** |  |  |  |  | **2%** |
| ***ZMYM3*** | **2%** | **2%** | **2%** |  | **2%** |
| ***CDKN2A*** |  | **0%** | **0%** |  | **2%** |
| ***DDX3X*** | **2%** | **3%** | **3%** |  | **2%** |
| ***STAG2*** |  |  |  |  | **2%** |
| ***CEBPA*** |  |  |  |  | **2%** |
| ***GATA2*** |  |  |  |  | **2%** |
| ***KIT*** |  |  |  |  | **2%** |
| ***RUNX1*** |  |  |  |  | **2%** |
| ***SETBP1*** |  |  |  |  | **2%** |
| ***EGR2*** | **3%** | **2%** | **3%** |  | **2%** |
| ***TCF12*** |  |  |  |  | **2%** |
| ***SCRIB*** |  |  |  |  | **2%** |
| ***RB1*** |  |  |  |  | **2%** |
| ***ITPKB*** |  |  |  |  | **2%** |
| ***SAMHD1*** | **2%** |  | **1%** |  | **2%** |
| ***PRKD3*** |  |  |  |  | **2%** |
| ***BIRC3*** | **4%** | **9%** | **6%** | **2%** |  |
| ***MGA*** | **3%** | **3%** | **3%** |  |  |
| ***KRAS*** | **3%** |  | **1%** |  |  |
| ***FBXW7*** | **2%** | **1%** | **2%** |  |  |
| ***IGLL5*** | **2%** |  | **1%** |  |  |
| ***IKZF3*** | **2%** |  | **1%** |  |  |
| ***MAP2K1*** | **2%** |  | **1%** |  |  |
| ***IRF4*** | **2%** | **1%** | **2%** |  |  |
| ***NRAS*** | **2%** | **0%** | **1%** |  |  |
| ***NXF1*** | **2%** |  | **1%** |  |  |
| ***CARD11*** | **1%** |  | **1%** |  |  |
| ***DYRK1A*** | **1%** |  | **1%** |  |  |
| ***HIST1H1E*** | **1%** |  | **1%** |  |  |
| ***PTPN11*** | **1%** | **1%** | **1%** |  |  |
| ***XPO4*** | **1%** |  | **1%** |  |  |
| ***BRCC3*** | **1%** |  | **1%** |  |  |
| ***TRAF2*** | **1%** |  | **1%** |  |  |
| ***ASXL1*** | **1%** | **1%** | **1%** |  |  |
| ***CHEK2*** | **1%** |  | **1%** |  |  |
| ***EWSR1*** | **1%** |  | **1%** |  |  |
| ***GNB1*** | **1%** |  | **1%** |  |  |
| ***HISTIHIB*** | **1%** | **1%** | **1%** |  |  |
| ***PIM1*** | **1%** |  | **0%** |  |  |
| ***ZNF292*** |  | **5%** | **2%** |  |  |
| ***TRAF3*** |  | **3%** | **1%** |  |  |
| ***SETD2*** |  | **2%** | **1%** |  |  |
| ***BRAF*** |  | **2%** | **1%** |  |  |
| ***SYNE1*** |  | **2%** | **1%** |  |  |
| ***XPO1*** |  | **2%** | **1%** |  |  |
| ***ARID1A*** |  | **2%** | **1%** |  |  |
| ***ATRX*** |  | **2%** | **1%** |  |  |
| ***FSIP2*** |  | **2%** | **1%** |  |  |
| ***CCND2*** |  | **1%** | **1%** |  |  |
| ***CNOT3*** |  | **1%** | **1%** |  |  |
| ***NXF1*** |  | **1%** | **1%** |  |  |
| ***SPEN*** |  | **1%** | **1%** |  |  |
| ***KIAA0947*** |  | **1%** | **1%** |  |  |
| ***MLL2*** |  | **1%** | **1%** |  |  |
| ***NFKBIE*** |  | **1%** | **1%** |  |  |
| ***SETD1A*** |  | **1%** | **1%** |  |  |
| ***FUBP1*** |  | **1%** | **0%** |  |  |
| ***POLR3B*** |  | **1%** | **0%** |  |  |
| ***RPS15*** |  | **1%** | **0%** |  |  |
| ***ANKHD1*** |  | **1%** | **0%** |  |  |
| ***BAX*** |  | **1%** | **0%** |  |  |
| ***BAZ2A*** |  | **1%** | **0%** |  |  |
| ***CREBBP*** |  | **1%** | **0%** |  |  |
| ***DNAJC11*** |  | **1%** | **0%** |  |  |
| ***IKZF3*** |  | **1%** | **0%** |  |  |
| ***KRAS*** |  | **1%** | **0%** |  |  |
| ***LUC7L2*** |  | **1%** | **0%** |  |  |
| ***SKIV2L2*** |  | **1%** | **0%** |  |  |
| ***ZC3H18*** |  | **1%** | **0%** |  |  |
| ***MED1*** |  | **0%** | **0%** |  |  |
| ***NKAP*** |  | **0%** | **0%** |  |  |
| ***TLR2*** |  | **0%** | **0%** |  |  |
| ***CD79A*** |  | **0%** | **0%** |  |  |
| ***CD79B*** |  | **0%** | **0%** |  |  |
| ***CDKN1B*** |  | **0%** | **0%** |  |  |
| ***IRAK1*** |  | **0%** | **0%** |  |  |

***Mean percentage of data proposed Landau et al.(2015) and Puente et al.(2015)**

**† Sanger sequencing was performed in this study**

**References**

1. Landau DA, Tausch E, Taylor-Weiner AN, Stewart C, Reiter JG, Bahlo J, et al. Mutations driving CLL and their evolution in progression and relapse. Nature. 2015;526(7574):525-30. doi: 10.1038/nature15395. Epub 2015 Oct 14.

2. Puente XS, Bea S, Valdes-Mas R, Villamor N, Gutierrez-Abril J, Martin-Subero JI, et al. Non-coding recurrent mutations in chronic lymphocytic leukaemia. Nature. 2015;526(7574):519-24. doi: 10.1038/nature14666. Epub 2015 Jul 22.

3. Xia Y, Fan L, Wang L, Gale RP, Wang M, Tian T, et al. Frequencies of SF3B1, NOTCH1, MYD88, BIRC3 and IGHV mutations and TP53 disruptions in Chinese with chronic lymphocytic leukemia: disparities with Europeans. Oncotarget. 2015;6(7):5426-34.
