# Supplementary material for: Genomic Profile of Chronic Lymphocytic Leukemia in Korea Identified by Targeted Sequencing
Source: PLoS One. 2016 Dec 13;11(12):e0167641. doi: 10.1371/journal.pone.0167641 (PMC5154520; doi:10.1371/journal.pone.0167641)
Supplement: S7 Table — (DOCX) [file pone.0167641.s007.docx]

**S7 Table. Mutation percentage difference between Caucasians and Koreans**

| **Gene** | **Number of patients in Caucasians**  **(n=990) ^*^** | **Mutation Frequency (%)** | **Number of patients in Koreans**  **(n=48)** | **Mutation Frequency (%)** | **Mutation Frequency Percentage Difference between  Koreans and Caucasians**  **(Koreans-Caucasians)** | ***P*-value^†^** | **P-values after FDR correction for multiple comparisons** | **95% CI** | |
| --- | --- | --- | --- | --- | --- | --- | --- | --- | --- |
|  |  |  |  |  |  |  |  | **Lower** | **Upper** |
| ***ATM*** | **132** | **13.33%** | **10** | **20.83%** | **0.075** | **0.140** | **0.420** | **-0.025** | **0.175** |
| ***TP53*** | **63** | **6.36%** | **7** | **14.58%** | **0.082** | **0.037** | **0.278** | **0.010** | **0.155** |
| ***SF3B1*** | **153** | **15.45%** | **5** | **10.42%** | **-0.050** | **0.343** | **0.837** | **-0.154** | **0.054** |
| ***KLHL6*** | **14** | **1.41%** | **4** | **8.33%** | **0.069** | **0.008** | **0.120** | **0.031** | **0.107** |
| ***BCOR*** | **18** | **1.82%** | **3** | **6.25%** | **0.044** | **0.069** | **0.338** | **0.004** | **0.085** |
| ***NOTCH1*** | **99** | **10.00%** | **3** | **6.25%** | **-0.038** | **0.617** | **0.837** | **-0.124** | **0.049** |
| ***MYD88*** | **34** | **3.43%** | **2** | **4.17%** | **0.007** | **0.681** | **0.837** | **-0.046** | **0.060** |
| ***POT1*** | **49** | **4.95%** | **1** | **2.08%** | **-0.029** | **0.725** | **0.837** | **-0.091** | **0.033** |
| ***CHD2*** | **54** | **5.45%** | **1** | **2.08%** | **-0.034** | **0.509** | **0.837** | **-0.099** | **0.031** |
| ***MED12*** | **15** | **1.52%** | **1** | **2.08%** | **0.006** | **0.534** | **0.837** | **-0.030** | **0.041** |
| ***ZMYM3*** | **19** | **1.92%** | **1** | **2.08%** | **0.002** | **0.616** | **0.837** | **-0.038** | **0.041** |
| ***CDKN2A*** | **1** | **0.10%** | **1** | **2.08%** | **0.020** | **0.090** | **0.338** | **0.007** | **0.033** |
| ***DDX3X*** | **25** | **2.53%** | **1** | **2.08%** | **-0.004** | **1.000** | **1.000** | **-0.050** | **0.041** |
| ***EGR2*** | **26** | **2.63%** | **1** | **2.08%** | **-0.005** | **1.000** | **1.000** | **-0.052** | **0.041** |
| ***SAMHD1*** | **11** | **1.11%** | **1** | **2.08%** | **0.010** | **0.435** | **0.837** | **-0.021** | **0.041** |

***Sum of data proposed at Landau et al.(2015) and Puente et al.(2015)**

**^†^ *P*-values calculated by Chi-square test or Fisher's exact test**
