# Supplementary material for: Genomic Profile of Chronic Lymphocytic Leukemia in Korea Identified by Targeted Sequencing
Source: PLoS One. 2016 Dec 13;11(12):e0167641. doi: 10.1371/journal.pone.0167641 (PMC5154520; doi:10.1371/journal.pone.0167641)
Supplement: S8 Table — (DOCX) [file pone.0167641.s008.docx]

**S8 Table. Incidence (%) of chromosomal abnormalities in patients with CLL (previous and present studies)**

|  | **Wu et al. [1],** | **Dong et al. [2],** | **Döhner et al. [3]** | **Chena et al. [4]** | **Glassman and Hayes [5]** | **Grever et al. [6],** | **Chang et al. [7]** | **Yoon et al. [8]** | **present study** |
| --- | --- | --- | --- | --- | --- | --- | --- | --- | --- |
| **Aberration** | **Taiwan (n=83)** | **China (n=173)** | **Germany (n=325)** | **Argentina (n=57)** | **USA**  **(n=100)** | **USA (n=235)** | **Korea (n=16)** | **Korea (n=48)** | **Korea (n=72)** |
| **13q14 deletion** | **45.8** | **27.7** | **55** | **63.2** | **40** | **34.0** | **69** | **12.5** | **45.8** |
| **Trisomy 12** | **20.5** | **21.9** | **16** | **35** | **11** | **20.4** | **19** | **29.1** | **30** |
| **17p deletion** | **10.8** | **7.5** | **7** | **11** | **12** | **8.1** | **0** | **10.4** | **23.5** |
| **11q deletion** | **13.3** | **10.9** | **18** | **ND** | **23** | **17.0** | **14** | **12.5** | **18.2** |
| **By conventional chromosome analysis** | | | | | | | | | |
| **Clonal abnormalities** | **42.2** | **NA** | **NA** | **48.8** | **28** | **NA** | **15** | **25.5** | **28** |
| **Normal karyotype** | **57.8** | **NA** | **NA** | **51.2** | **72** | **NA** | **85** | **74.5** | **65** |
| **By fluorescence *in situ* hybridization** | | | | | | | | | |
| **Clonal abnormalities** | **69.9** | **NA** | **82** | **80.7** | **64** | **84.3** | **63** | **52** | **66.7** |
| **Normal karyotype** | **30.1** | **NA** | **18** | **19.3** | **36** | **15.7** | **37** | **48** | **33.3** |
| **Cytogenetic methods** | **conventional cytogenetic studies** | **Conventional cytogenetic studies** | **NA** | **Conventional cytogenetic studies** | **Conventional cytogenetic studies** | **NA** | **Conventional cytogenetic studies** | **Conventional cytogenetic studies** | **Conventional cytogenetic studies** |
| **B cell stimulator** | **12-O-tetradecanoyl phorbol-13-**  **acetate** | **interleukin 2 and CpG-oligonucleotide DSP30** | **NA** | **pokeweed mitogen,**  **and lipopolysaccharide** | **lipopolysaccharide (LPS) mitogen** | **NA** | **NA** | **Lipopolysaccharide** | **TPA** |
| **FISH** | **done** | **done** | **done** | **done** | **done** | **done** | **done** | **done** | **done** |

**^NA; not applicable^**

**References**

1. Wu SJ, Lin CT, Huang SY, Lee FY, Liu MC, Hou HA, et al. Chromosomal abnormalities by conventional cytogenetics and interphase fluorescence in situ hybridization in chronic lymphocytic leukemia in Taiwan, an area with low incidence--clinical implication and comparison between the West and the East. Ann Hematol. 2013;92(6):799-806. doi: 10.1007/s00277-013-1700-x. Epub 2013 Feb 17.

2. Dong HJ, Zhou LT, Zhu DX, Wang DM, Fang C, Zhu HY, et al. The prognostic significance of TP53 mutations in Chinese patients with chronic lymphocytic leukemia is independent of del(17p13). Ann Hematol. 2011;90(6):709-17. doi: 10.1007/s00277-010-1125-8. Epub 2010 Nov 27.

3. Döhner H, Stilgenbauer S, Benner A, Leupolt E, Kröber A, Bullinger L, et al. Genomic aberrations and survival in chronic lymphocytic leukemia. New England Journal of Medicine. 2000;343(26):1910-6.

4. Chena C, Arrossagaray G, Scolnik M, Palacios MaF, Slavutsky I. Interphase cytogenetic analysis in Argentinean B-cell chronic lymphocytic leukemia patients: association of trisomy 12 and del (13q14). Cancer genetics and cytogenetics. 2003;146(2):154-60.

5. Glassman AB, Hayes KJ. The value of fluorescence in situ hybridization in the diagnosis and prognosis of chronic lymphocytic leukemia. Cancer genetics and cytogenetics. 2005;158(1):88-91.

6. Grever MR, Lucas DM, Dewald GW, Neuberg DS, Reed JC, Kitada S, et al. Comprehensive assessment of genetic and molecular features predicting outcome in patients with chronic lymphocytic leukemia: results from the US Intergroup Phase III Trial E2997. J Clin Oncol. 2007;25(7):799-804. Epub 2007 Feb 5.

7. Chang YH, Park J, Kim HC, Chun HK, Kim YR, Kim M, et al. Korean patients with chronic lymphocytic leukemia show the similar types of chromosomal aberrations as those in Europe and North America. Leuk Res. 2006;30(6):695-9. doi: 10.1016/j.leukres.2005.10.013. PubMed PMID: 16297977.

8. Yoon JH, Kim Y, Yahng SA, Shin SH, Lee SE, Cho BS, et al. Validation of Western common recurrent chromosomal aberrations in Korean chronic lymphocytic leukaemia patients with very low incidence. Hematol Oncol. 2014;32(4):169-77. doi: 10.1002/hon.2103. Epub 013 Oct 7.
